# Carbonyl–Olefin/Alkyne Metathesis Reactions Catalyzed by Bifunctional H‑USY Zeolites

**DOI:** 10.1021/jacs.5c11880

**Published:** 2025-08-27

**Authors:** Paloma Mingueza-Verdejo, David Velázquez-Ojeda, Cristina Bilanin, Francisco Garnes-Portolés, Silvia Rodríguez-Nuévalos, Raúl Pérez-Ruiz, Judit Oliver-Meseguer, Antonio Leyva-Pérez

**Affiliations:** † Instituto de Tecnología Química (UPV−CSIC), Universitat Politècnica de València−Agencia Estatal Consejo Superior de Investigaciones Científicas, Avda. de los Naranjos s/n, 46022 Valencia, Spain; ‡ Departamento de Química, Universitat Politècnica de València, Avda. de los Naranjos s/n, 46022 Valencia, Spain

## Abstract

Metal-, organo-, and proton-catalyzed carbonyl–olefin/alkyne metathesis reactions have gained relevance in organic synthesis during the past decade, but their potential implementation in high-volume processes (i.e., in flow) is severely hampered by the lack of a general, robust, easily separable, and nontoxic solid catalyst. Both Brønsted and Lewis acid sites inside molecular-sized soluble cages seem to be involved during the catalytic process in solution; thus, a similar bifunctional acid solid catalyst, in a confined space, could play the desired catalytic role. We show here that commercially available, ultrastabilized aluminosilicate acid faujasites (H-USY zeolites), containing Brønsted and Lewis acid sites in microporous channels and cavities, catalyze a variety of intra- and intermolecular metathesis reactions between aldehydes/ketones and alkenes/alkynes, with diverse structural patterns, in reasonable yields and under mild reaction conditions. The zeolite can be easily recovered after the reaction in batch and reused or implemented in in-flow processes for continuous synthesis of the metathesis products. These results open the way to designing carbonyl–olefin/alkyne metathesis reactions with simple solid catalysts.

## Graphical abstract

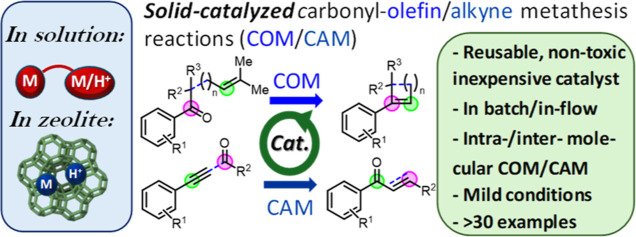

## Introduction

The carbonyl–olefin metathesis (COM) reaction^1^ holds tremendous synthetic potential at a high scale since carbonyl and alkene groups are arguably the most available functionalities in organic molecules, either natural or synthetic.^2^ The successful transitioning of the parent alkene cross-metathesis reaction from the laboratory to the industry (and vice versa)^3^ supports this assumption. Thus, it is not surprising that a research effort has been devoted during the past decade in the pursuit of the best catalysts for the COM reaction,^4^ dramatically expanding the precedent knowledge on this reaction from the photochemistry field (Paterno–Büchi reaction).^5^ Figure 1 summarizes the soluble metal-,^6^ organo-,^7^ and proton^8^ catalysts reported for the COM reaction; however, most, if not all, of them are unrecoverable after the reaction. Only two examples of solid catalysts for the COM reaction can be found in the literature,^9^ as far as we know, and both of them report particular clays (montmorillonite types) as solid catalysts for selected metathesis reactions, with a defined structural pattern in the reactants and little room for diversification. This structural constraint also occurs for most of the reported homogeneous catalysts,^4,6–8^ which, despite the merit of catalyzing such challenging metathesis reactions, suffer from severe selectivity issues when going from intra- to intermolecular COM versions or even after slight variations in the reactants’ carbon skeletons.^1,10^ Thus, in order to consider a potential sustainable industrial implementation of the COM reaction, it seems mandatory to find a solid catalyst which is not only cheap, robust, and easily recoverable but also efficiently catalyzes the reaction with structural diversity and can be engineered in in-flow processes.^11^ The translation between soluble and solid catalysts was indeed key in the development of the parent alkene cross-metathesis reaction,^3d^ and this step for the COM reaction would allow telescoping the reaction with other catalytic organic transformations.^12^

**Figure 1:**
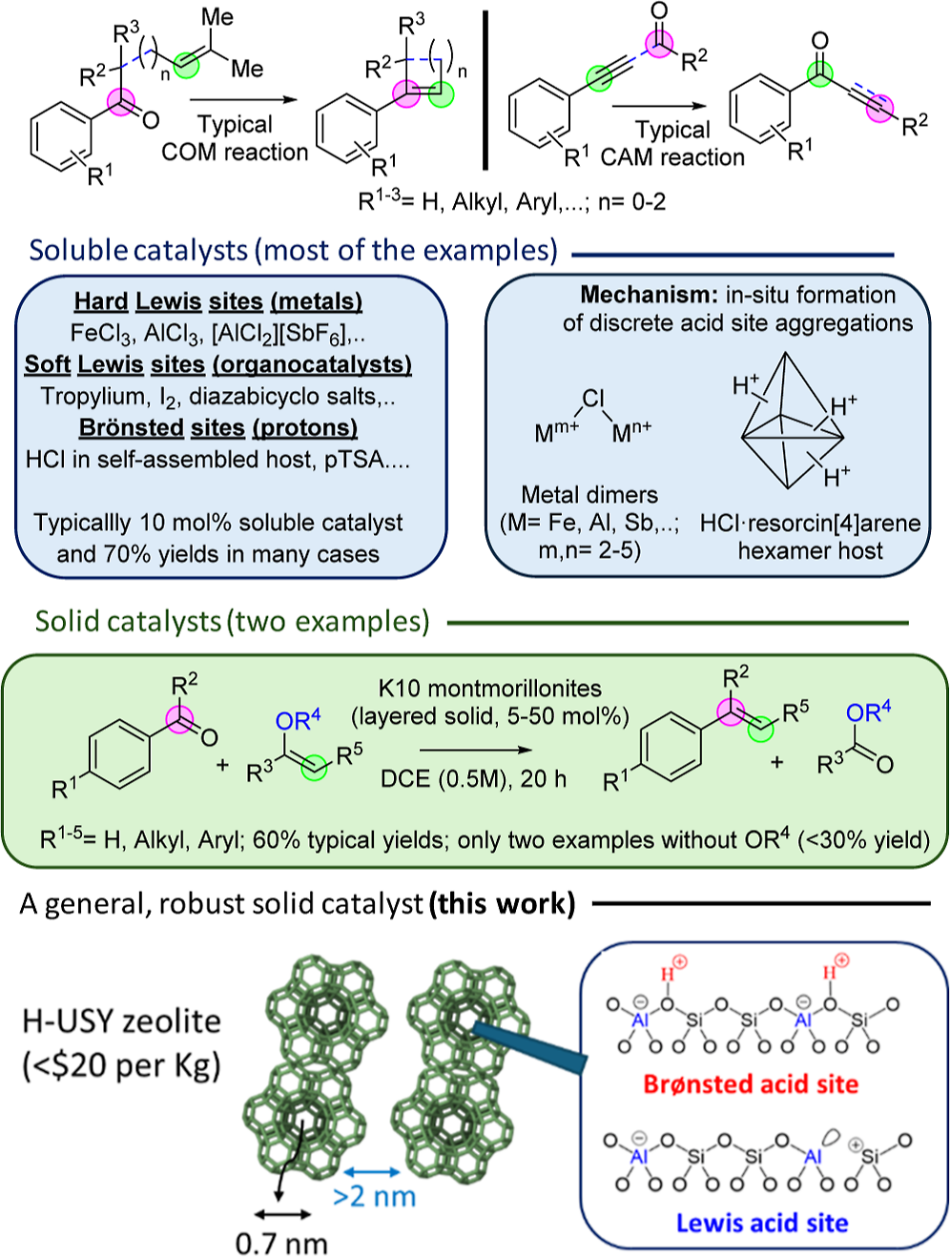
Typical COM and CAM reactions, with representative reported catalysts and yields, and a parallelism between the aggregated catalytic sites of soluble catalysts and the H-USY zeolite. The dotted blue lines represent the intra- and intermolecular metathesis versions.

Figure 1 also shows that either strong (i.e., Fe^3+^) or soft (i.e., carbocations) Lewis acid-soluble compounds and, more recently, simple Brønsted acids (i.e., *para*-toluenesulfonic acid, *p*TSA) catalyze the COM reaction in its different intra- and intermolecular versions, and this fact strongly suggests that a combination of both types of acid sites could be involved during the catalytic process. Mechanistic studies have revealed that discrete aggregations of acid sites in solution,^13^ for instance, of two Fe^3+^ cations^13a^ or several H^+^ sites,^9a,9b^ are responsible for catalysis; thus, we envisioned that a suitable solid acid containing acid sites in a molecular-sized confined space (in contrast to montmorillonite clays with ionic layers) may catalyze the COM reaction in a more general way.^14^ The requirement of two adjacent metal sites during the catalysis reaction has also been recently reported for the challenging carbonyl–carbonyl cross-metathesis reaction.^15^ The rationale above is also valid for the more recently developed carbonyl–alkyne metathesis (CAM) reaction;^16^ indeed, a common solid catalyst for both COM and CAM reactions would bridge the gap between these two related transformations.

Here, we show that ultrastabilized H–Y zeolites (H-USYs, see Figure 1) catalyze the intra- and intermolecular COM and CAM reactions with structural diversity in the reactants. These zeolites are massively employed in petrochemistry and are thus cheap and highly available. Structurally, H-USY zeolites are 3D crystalline microporous aluminosilicates which, after a dealumination treatment, generate Brønsted and Lewis sites together with mesopores to further stabilize the microporous structure.^17^ The H-USY zeolitic framework is extraordinarily robust (calcination at >600 °C without deterioration) and allows the diffusion of typical reactants in the COM and CAM reactions to the catalytic sites, to potentially mimic the in situ-aggregated sites found in soluble acid catalysts.^17a^ The thermal robustness of the zeolite may seem useless for the mild conditions of this organic reaction; however, it would become crucial for reusing (see ahead). In addition, in practical terms, H-USY zeolites are commercially available at inexpensive prices (<$20 per kg), in different acid forms, and zeolites are generally nontoxic (employed in household materials).

## Results and Discussion

Table 1 shows the catalytic results for the intramolecular COM reaction of ketoester **1a**, a typical starting material for this reaction, in the presence of different solid acids. The synthesis and characterization of the starting materials and products can be found in Section 1 of the Supporting Information (S1), and the physicochemical parameters of the different solid acids, including Brønsted and Lewis acid content sites, crystallinity, and surface area, among others, are described in Section 2 (Supporting Information, S2). The reactions were performed in a sealed vial placed in a preheated oil bath at 70 °C under magnetic stirring, using dichloroethane as a solvent (DCE, 0.25 M) and measuring the reaction mixture during 24 h reaction time. These reaction conditions were selected after optimizing the same COM reaction with FeCl_3_ as a catalyst (see Supporting Information, S3), and these conditions are in good agreement with the reported ones. A 10 wt % of solid catalyst was initially employed, which corresponds to ≈2 mol % acid sites for the H-USY zeolite (Si/Al = 15) according to Fourier transform infrared (FT-IR) experiments, pyridine titrations, and temperature-programmed ammonia desorption (TPD) analyses (Figures S7–S9 and Table S3), which accounts for a catalytic amount not only lower than FeCl_3_ (Figures S12–S13) but also of many soluble catalysts employed for the COM reaction (Table S13). Notice that for zeolites with a higher Si/Al ratio, the mol % of acid sites in the reaction is lower.

**Table 1:**
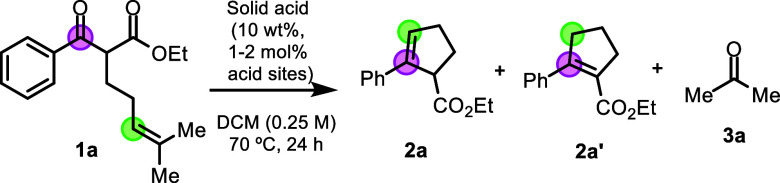
Catalytic Results for the Intramolecular COM Reaction of **1a** under the Indicated Reaction Conditions

entry	catalyst (Si/Al ratio)	yield of 2a (%)^a^
1	none	
2^b^	H-USY zeolite (15)	27
**3** ^c^	**H-USY** **zeolite (15)**	**84 [58]**
4	Acid Al_2_O_3_	
5	SiO_2_–Al_2_O_3_	6
6	MCM–41 (40)	5
7	H-Beta zeolite (15)	5
8	H-ZSM5 zeolite (15)	4
9	H-mordenite	15
10	H-USY zeolite (20)	81
**11**	**H-USY** **zeolite (28)**	**>99**
12	H-USY zeolite (40)	32
13	Fe^3+^–NaY zeolite	

a Triple-checked by gas chromatography coupled to mass spectrometry (GC–MS), ^1^H nuclear magnetic resonance (^1^H NMR), and isolated weight and reproduced twice (average yield).

b Nondehydrated sample.

c Reused after washing with hexane and drying under vacuum at 250 °C.

The results show that the COM product **2a** is obtained in 84% yield with the previously dehydrated (250 °C under vacuum) H-USY zeolite catalyst (Si/Al = 15), in contrast to the noncatalyzed reaction or the nondehydrated zeolite (entries 1–3). The dehydration procedure releases the Lewis zeolitic acid sites after water removal, allowing for better coordination of the incoming reactants, **1a** in this case. A hot filtration test clearly indicates that the catalytic events occur onto the solid, since any catalytically active species is not detected in solution (see Supporting Information, S4, Figure S14). The zeolite could be recovered by centrifugation and reused; however, the yield of **2a** was <20%, even after further dehydration. Exhaustive washings of the used zeolite by Soxhlet extraction did not improve the yield after reuse; however, calcination at 500 °C of the used zeolite significantly restored its catalytic activity (58% final yield of **2a**, entry 3 in Table 1). The H-USY zeolite turns from a violet color, which belongs to the typical polycondensated high-weight byproducts formed from activated carbonyl compounds (including the byproduct acetone **3a**) along the COM reaction,^18^ here entrapped inside the pores,^19^ to a colorless solid after calcination at 500 °C (not after dehydration at 250 °C under vacuum), which showcases the relevance of having a thermally robust solid catalyst to burn away the undesired byproducts formed during the COM reaction. In this way, the catalytic H-USY zeolite could be reused up to six times without any depletion of the catalytic activity (Figure S15). Moreover, kinetic experiments conducted at different stirring velocities indicate that the initial rate is not controlled by diffusion through the zeolite channels, as v_o_ remains relatively constant between 300 and 1200 rpm, and the final conversions obtained range from 71% to 81% (Figure S24). One-dimensional micropore zeolites, such as H-mordenite (entry 9), gave small yields of **2a** but higher yields than those of other micropore structures such as H-Beta and H-ZSM-5. To obtain the carbon balance of the reaction, the zeolite was filtered from the reaction medium, and the resulting solution was concentrated under vacuum; the zeolite was washed using the Soxhlet method, with DCM at 70 °C overnight, and then extracted with water, and the resulting organic phases were concentrated under vacuum again. The crude reaction products obtained from the two phases were mixed and purified by column chromatography. The total mass obtained was 119.1 mg, corresponding to a yield of 89.4% of the purified product, 19.3% of which was obtained by washing the zeolite with a Soxhlet and the remaining 80.7% from the solution. These numbers are quite reasonable for a very good mass balance of the reaction.

Nonstructured acid alumina and silico-alumina solids gave none or very minor amounts of product **2a** (0% and 6%; entries 4 and 5, respectively), while the 1D mesoporous AlMCM-41 (pore size ≈20 Å) and the 3D purely microporous H-Beta (pore size ≈7 Å) and H-ZSM-5 (pore size ≈5.5 Å) zeolites also gave minor amounts of **2a** (≈5%, entries 6–8). These minor yields of **2a** were also found with the organic acid solid Amberlyst (see Supporting Information, S5). Despite the fact that both H-USY and H-Beta zeolites have micro- and mesoporosity as well as both Lewis and Brønsted acid sites, only H-USY exhibited good activity, showcasing the subtility of the COM reaction on solid acid catalysts, also demonstrated by the very different catalytic activities of H-USY zeolites with different Si/Al ratios (see ahead). These results strongly suggest the necessity of having a combination of micro- and mesopores in the zeolite, together with the suitable structure and Si/Al ratio, to catalyze the COM reaction, which occurs better in the H-USY rather than in the H-Beta zeolite. Thus, at this point, we tested other H-USY zeolites with different Si/Al ratios, between 20 and 40 (entries 10–12), and a quantitative yield of **2a** (>99%) was found for the H-USY zeolite with intermediate acidity (Si/Al = 28). Comparative kinetic experiments (S5 and Figure S19) confirm the catalytic superiority of the latter. It is noteworthy to comment here that the higher the H-USY zeolite Si/Al ratio, the stronger the acid sites and the higher the number of mesopores (see Supporting Information, S2), which confirms that a balance between acidity and mesopores is required for this COM reaction. When we try to correlate the initial rate for the COM reaction with the total amount of Brønsted acid sites (obtained by pyridine titration) of the zeolite catalyst, the tendency does not follow a straightforward line, but it does when we correlate the initial rates obtained with the mmol of Brønsted acid sites obtained after pyridine desorption at 150 °C (closer to the reaction conditions), which indicates that the weaker Brønsted acid sites are responsible for the catalysis reaction (together with Lewis acid sites, see ahead), and it is the amount of them rather than the amount of total acid sites that controls the catalytic reaction, which explains the lack of a clear tendency for the zeolites’ Si/Al ratio. An additional advantage of the zeolite-catalyzed COM reaction is that byproduct **2a**′, recurrently found with acid-soluble catalysts after alkene isomerization in **2a** (Table S5), was found here in <1% in all cases, due to the mild acidity of the zeolite compared to, for instance, sulfonic acids.

A potential catalysis by metal impurities in the zeolite was discarded on the basis of analytical and reactive results. The inductively coupled plasma-atomic emission spectroscopy (ICP-AES) results for 13 different metals show that, apart from Al, only Fe is present as a metal in significant amounts in the H-USY zeolite (Si/Al = 15), 20 times less (≈150 ppm) than Al (>30,000 ppm, Table S14). In addition, the use of the corresponding Fe^3+^-containing Na–Y zeolite, prepared by cation exchange, did not produce any conversion of **1a** (entry 13 in Table 1).

The intermolecular COM reactions of either naphthaldehyde **4a** or benzaldehyde **4b** with 2-methylbut-2-ene **5a** (see Supporting Information, S6) was studied under optimized reaction conditions, and the intermolecular CAM reaction of benzaldehyde **4b** with phenylacetylene **9** (see Supporting Information, S7) was also studied. In all cases, the dehydrated H-USY zeolite (Si/Al = 28) showed the best catalytic behavior with moderate-to-good yields of the desired metathesis products. The typical byproducts observed for the intermolecular COM reaction are coupled alkene products (Figure S16). The zeolite catalysts could be recovered and reused five times for the intermolecular CAM reaction (Figure S17), without significant depletion of the catalytic activity. Since acetophenone **11** was produced during the CAM reaction, molecular sieves or the reaction after several cycles on N_2_–vacuum to reduce as much as possible the presence of water in the reaction medium, were tested, but acetophenone **11** continues forming. We have also characterized the solid after the reaction and observed that the band of absorbed water disappears after the first cycle, so water has to be present either in the reactants or in the solvent after use. Karl–Fischer analyses were carried out, and the amount of water was: 0.0138% for DCE, 0.0787% for phenylacetylene **9,** and 5.48% for benzaldehyde **4b**. With these results in hand, a new commercial sample of dried benzaldehyde **4b** was purchased (with any water absent) and used to see if the formation of acetophenone **11** could be slowed down. However, it does not occur, since it seems that the minimum presence of water is enough to form acetophenone **11**, i.e., the hydration reaction is highly favored. In any case, phenylacetylene **9** is in excess and does not compromise the reaction yield for the limiting reactant during the CAM reaction.

Figure 2 shows that not only the optimized reactions but also other intra- and intermolecular COM and CAM reactions occur in the presence of the catalytic H-USY (Si/Al = 28) zeolite. The intramolecular COM reaction gave moderate-to-excellent isolated yields for different types of cyclopentenes (**2a**–**2g**, 49–>99%) and naphthalenes (**2h**–**2i**, 53–>99%) with different substituents (methoxy and bromide), although a low yield of cyclohexene **2j** (11%) was achieved. This result shows that, although the H-USY zeolite reacts with a variety of substrates, some of them can still be quite unreactive. Nevertheless, it must be said here that there are just a couple of catalysts in the literature capable of catalyzing both intramolecular and intermolecular COM reactions (all of them soluble and unrecoverable),^4^ but the H-USY (Si/Al = 28) zeolite was also catalytically active for the intermolecular COM reaction between benzaldehydes containing methoxy, chloride, and fluoride substituents (**4a**–**4h**) and 2-methylbut-2-ene (products **6aa–6ha**), or 5-bromo-2-methylpent-2-ene (products **6ab–6bc**), with moderate to low isolated yields (up to 49%). In all cases, the major isomer obtained for the intermolecular COM reaction is the *E* product, except for **6fa**, where an equimolar *Z*/*E* mixture was observed. The product yields, although they seem low in some cases, are not far from those with homogeneous catalysts (see Supporting Information, S8), which emphasizes the remarkability of the zeolite catalyst.

**Figure 2:**
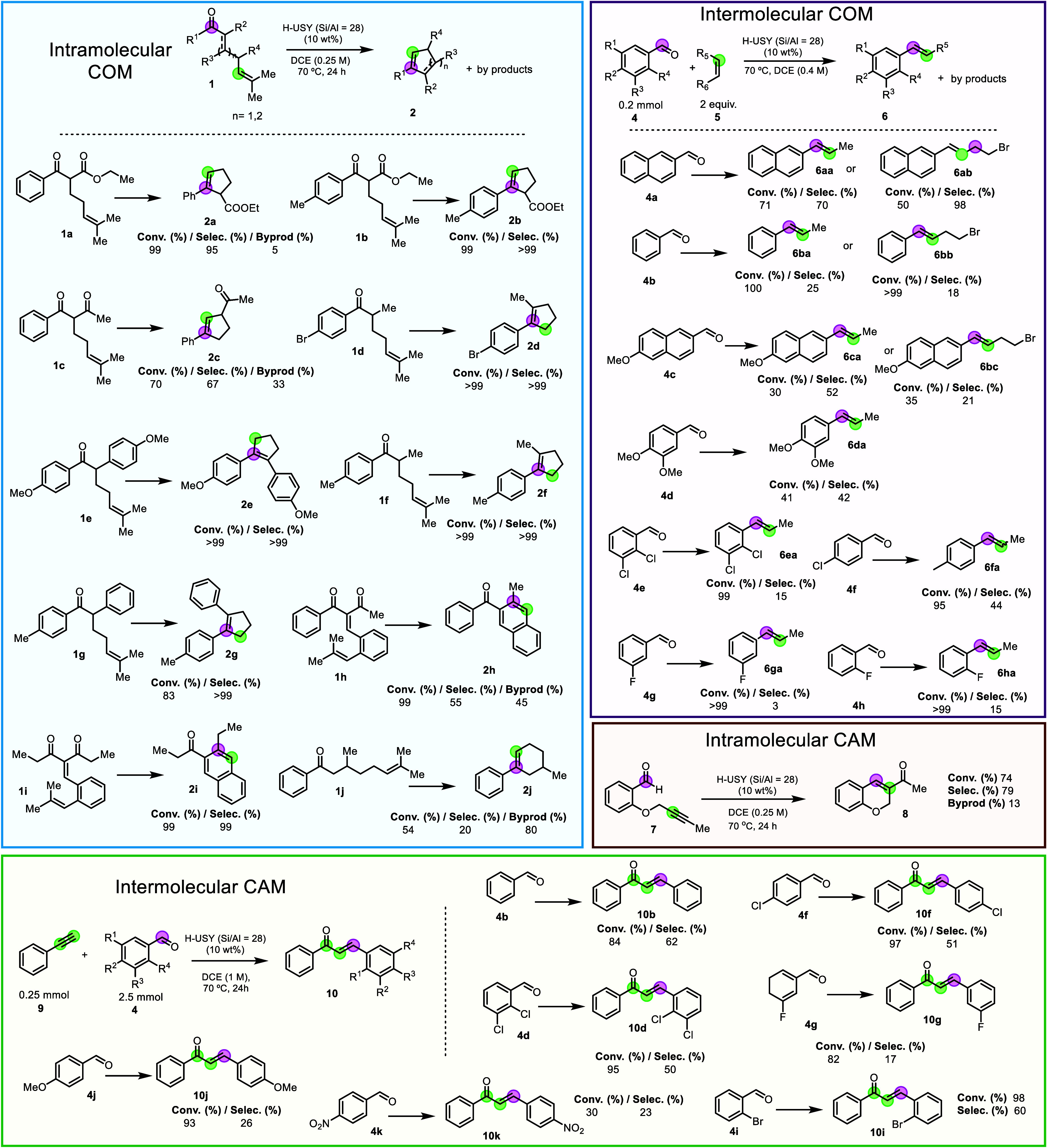
Scope of different COM and CAM reactions catalyzed by the H-USY zeolite (Si/Al = 28) under the indicated reaction conditions. Average yield of two reproductions.

Not only the intramolecular COM but the intramolecular CAM reaction of alkyne **7** is also catalyzed by the H-USY zeolite, indeed, with a good isolated yield (59%) of product **8**. In the case of intermolecular CAM reactions, the reaction of benzaldehydes **4b–4k** with phenylacetylene **9** gave the chalcone products **10b**–**10j**, containing methoxy, chloride, and fluoride substituents. The nitro-substituted product **10k** resulted in a very low isolated yield after the low conversion of nitrobenzaldehyde **4k**.

As commented above, a key step to scale-up an organic process is the implementation of an in-flow process. Indeed, the alkene–alkene metathesis reaction is massively performed in industry in continuous reactors. The results for the in-flow COM reaction of **1a** in a fixed-bed tubular reactor filled with the H-USY zeolite (Si/Al = 28) show that when a flow rate of 0.03 mL·min^–1^ was passed over the zeolite at 70 °C, complete conversion of **1a** with a selectivity of ∼60% to the product **2a** was observed, with 40% of the isomerized product **2a′** (see S6 and Figure S19). Since **2a′** could be formed due to the slow flow employed, which favors the isomerization of product **2a** inside the tubular reactor, the flow rate was increased to 0.05 mL·min^–1^, and Figure 3 shows that a product selectivity to **2a** of ∼90% was achieved, with still complete conversion of **1a**, which corresponds to 2.6 g of neat product **2a** at the exit of the tubular reactor. These results demonstrate that the zeolite catalyzes, very selectively and efficiently, the intramolecular COM reaction in continuous mode.^4g^ The reuses in the batch of the different H-USY zeolites also confirm the observed robustness (Figure S22).

**Figure 3:**
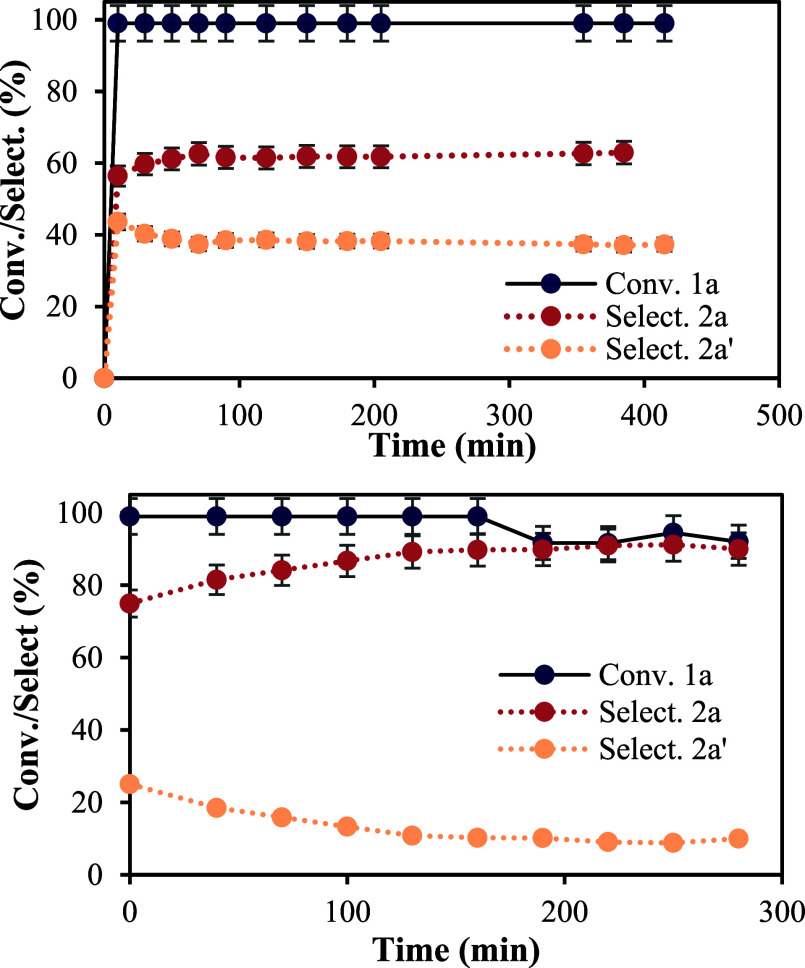
In-flow COM reaction of **1a** catalyzed by the H-USY zeolite (Si/Al = 28) at room temperature. Flow velocity: 0.03 and 0.05 mL·min^–1^, respectively. Error bars account for 5% uncertainty.

The nature of the catalytic sites in the H-USY zeolite was investigated through reactivity studies, kinetic experiments, and in situ NMR analysis (see Supporting Information, S2 and S10). To determine which type of acidity is responsible for catalytic activity, we conducted the carbonyl–olefin metathesis (COM) reaction of substrate **1a** under conditions where only Brønsted or Lewis acid sites were available. Using the commercially available Na–Y zeolite, which lacks Brønsted acidity due to the presence of Na^+^ instead of H^+^ ions, resulted in any conversion observed for **1a**. Conversely, treatment of H-USY (Si/Al = 28) with an ammonium fluorosilicate solution reduced the number of Lewis acid sites and increased the Si/Al ratio to over 100 (also enhancing mesoporosity; see Figures S3 and S10), but this modified zeolite was also inactive in the COM reaction (Figure S25). Together, these results demonstrate that both Brønsted and Lewis acid sites are essential for the catalytic activity in this system. In fact, if we relate the amount of pyridine per gram of catalysts calculated at 150 °C by FT-IR spectra pyridine desorption with the initial rates obtained, we observe a clear lineal relation for both Brønsted and Lewis acidity (Figure S21). Although the mechanism for the Brønsted acid-catalyzed reaction should be different from that of the Lewis acid-catalyzed reaction, the difference is difficult to distinguish since both sites act concomitantly during the reaction. The adjacent Brønsted and Lewis sites catalyze the reaction,^19b–19d^ thus enabling zeolites with higher Si/Al content to still be active for the reaction. The benefits of the H-USY hierarchical zeolitic structure for the COM reaction were assessed by kinetic experiments at different stirring speeds (Figure S24; see other comments above), which show that the reaction rate of **1a** does not change with stirring, indicating that external mass transfer limitations are absent in the micro- and mesopore zeolite frameworks. These results, together with the lack of catalytic activity of the microporous zeolites H-Beta and H-ZSM5 (see Table 1 above), strongly support the need for additional mesopore channels to manage the molecular traffic during the COM reaction. Notice that a H-Y zeolite (not ultrastabilized) is not stable enough to be tested in a control experiment under the COM reaction conditions.

The role of water was then examined. As commented above, the removal of chemisorbed water in the zeolite is essential for the catalytic activity, which supports the key role of the zeolite Lewis acid sites during the COM reaction.^20^ This makes sense considering the low nucleophilicity of the reactive functional groups, i.e., carbonyl and alkene, which are in the same range as H_2_O. In contrast, the exhaustive drying of the reaction mixture is not needed; thus, it seems that physisorbed water does not hamper the COM reaction. To check this, the dehydrated H-USY zeolite was allowed to stand at open ambient conditions for 1 week, and its catalytic activity was measured each day by kinetic experiments (Figure S26), without observing any significant rate variation. FT-IR measurements of the H-USY zeolite show that any strongly adsorbed water did not appear during the whole week of study (Figure S27), and these results together indicate the consistent catalytic activity of the H-USY zeolite, regardless of the presence or absence of water in the reaction medium (in reasonable amounts), which has evident positive practical implications. In the case of intermolecular CAM reaction, exhaustive drying of the zeolite may be needed in order to avoid acetophenone **11** as a byproduct. FT-IR analysis of the zeolite was performed before and after using it for catalyzing the corresponding reaction, and a decrease in the band that corresponds to absorbed water in the zeolite was noticed, but acetophenone still appeared as a byproduct. To assess where water was coming from, we also performed a Karl–Fischer experiment and analyzed the water present in both reactants and solvent, observing that both benzaldehyde and phenylacetylene had water (54752.9 and 786.5 ppm, respectively), which explains the appearance of acetophenone even when using cycles of vacuum–nitrogen before the reaction. Also, using new dried benzaldehyde or molecular sieves does not improve the result, which makes us think that little amount of water from the air is enough to form the acetophenone under reaction conditions.

The mechanism of the intermolecular COM and CAM reactions catalyzed by the H-USY zeolite was also studied by isotopic experiments (Supporting Information, S10). For the COM reaction, isotopically labeled ^18^O-benzaldehyde (^**18**^ **O–4b**) was reacted with 2-methylbut-2-ene **5a**, and the corresponding ^**18**^ **O-3** acetone byproduct was observed by GC–MS. For the CAM reaction, the same experiment was performed but with phenylacetylene **9** as a coupling partner, observing that the mass of the corresponding product mainly corresponds to the chalcone product ^**18**^ **O-10b**, although some contribution of the nonlabeled product **10b** appears, which suggests that a certain exchange with water can occur during the reaction (Figure S29). Additionally, a reaction between the isotopically labeled terminal carbon atom ^13^C-phenylacetylene (^**13**^ **C–9**) and benzaldehyde **4b** was carried out, and the corresponding ^13^C NMR spectrum shows that the ^13^C atom in the chalcone product is exclusively in the internal alkene position; neither the ketone, nor the external alkene atoms are isotopically marked (Figure S30). Remarkably, we managed to prepare the corresponding oxetane from **1a** by a Paterno–Büchi reaction.^4e^ It must be observed here that this oxetane has not been prepared before as far as we know, despite it being frequently claimed the key COM reaction intermediate.^4b^ After its photosynthesis, HPLC separation, and characterization by CG-MS and FT-IR analysis, oxetane was directly used as a starting material for the COM reaction, employing the same reaction conditions as when starting with **1a**, and product **2a** was formed, as confirmed by GC–MS (Figures S31–S32). These results suggest that this intermediate is responsible for the metathesis reaction in the channels of the zeolite, and a proposed mechanism can now be provided (Figure S33). A comparative kinetic experiment between the CAM reactions of **9** and **4b** and the condensation reaction of acetophenone **11** and benzaldehyde **4b** confirms that the CAM reaction products were not mainly formed by an aldol condensation reaction (Figure S34). These results are in line with the established mechanisms for the intermolecular COM and CAM reactions, showcasing that the H-USY zeolite catalyst proceeds through recognizable metathesis pathways.

## Conclusions

In summary, we have shown here that a variety of COM and CAM reactions are catalyzed by commercially available H-USY zeolites, by virtue of the combination of Brønsted and Lewis catalytic sites present in the micro- and mesopores of the partially de-aluminated zeolite framework. The solid catalyst can be reused and implemented under in-flow reaction conditions, opening the way for a general heterogeneously catalyzed COM and CAM reaction procedures.

## Supplementary Material



## Notes

Experimental section, compound characterization, additional comments, and additional references (PDF)
