# Supplementary material for: Carbonyl–Olefin/Alkyne Metathesis Reactions Catalyzed by Bifunctional H‑USY Zeolites
Source: J Am Chem Soc. 2025 Aug 27;147(36):33256–63. doi: 10.1021/jacs.5c11880 (PMC12426927; doi:10.1021/jacs.5c11880)
Supplement: Supplementary file 1 [file ja5c11880_si_001.pdf]

SUPPORTING INFORMATION

Carbonyl-olefin/alkyne metathesis reactions catalyzed by bifunctional H-USY zeolites

Paloma Mingueza–Verdejo,[†] David Velázquez–Ojeda,[†] Cristina Bilanin,[†] Francisco Garnes–Portolés,[†] Silvia Rodríguez–Nuévalos,[†] Raúl Pérez-Ruiz,[‡] Judit Oliver–Meseguer*,[†] and Antonio Leyva–Pérez.*,[†]

[†]Instituto de Tecnología Química (UPV–CSIC), Universitat Politècnica de València–Agencia Estatal Consejo Superior de Investigaciones Científicas, Avda. de los Naranjos s/n, 46022 Valencia, Spain.

[‡]Departamento de Química, Universitat Politècnica de València, Avda. de los Naranjos s/n, 46022 Valencia, Spain.

Corresponding authors: joliverm@itq.upv.es, anleyva@itq.upv.es.

Phone: +34963877800; Fax: +349638 77809.

- S1. General considerations: starting materials, products and characterization 3
S1.1 Materials: 3
S1.2 Physical techniques: 3
S1.3 Reaction procedures: 4
S1.4 Synthesis of the starting materials: 6
S1.5 Synthesis of the oxetanes intermediates and reactivity 8
S1.6 Compound characterization. 10
- S2. Characterization of the solid acids 22
S2.1 Summary of physicochemical parameters. 22
S2.2 Brunauer, Emmett and Teller (BET) surface area. 23
S2.3 Powder X-ray diffraction (PXRD) 26
S2.4 FESEM and HRTEM. 28
S2.5 Acidity measurements. 31
S2.6 ^{27}Al nuclear magnetic resonance (NMR). 37
- S3. Optimization of the intramolecular COM reaction with $FeCl_3$ 38
- S4. Leaching, by-products and reusing of USY zeolites. 40
- S5. Catalytic results with other solid acids. 44
S5.1. Catalyst screening and optimization. 44
S5.2. Appearance of by-product **2´**. 53
- S6. Optimization of the intermolecular COM reaction. 54
S6.1. Catalyst screening for the intermolecular carbonyl-olefin metathesis. 54
S6.2. Solvent screening. 55
S6.3. Solvent amounts screening. 56
S6.4. Zeolite amount. 57
- S7. Optimization of the intermolecular CAM reaction. 58
- S8. Comparison of the results with reported reactions. 61
- S9. Nature of the catalytic sites. 62
- S10 Reaction Mechanism. 68
- Additional references. 74

S1. General considerations: starting materials, products and characterization

S1.1 Materials:

All reagents (≥97% purity) and solvents (≥99% purity) were purchased from Sigma Aldrich-Merck and used as received unless otherwise indicated. H-USY zeolites (CBV-720, CBV-740, CBV-760 and CBV-780) and H-Beta zeolite (CP-811) were purchased from Zeolyst. K-10 was purchased from Merck-Aldrich.

S1.2 Physical techniques:

^{1}H and ^{13}C nuclear magnetic resonance (NMR) spectra were recorded at room temperature on a 400 MHz spectrometer (Bruker Ascend 400) using the appropriate deuterated solvent.

Gas chromatographic analyses were performed in an instrument (Agilent 8860) equipped with a 30 m x 250 µm x 0.25 µm Agilent HP-50+ capillary column. *N*–dodecane was used as an external standard. GC–MS analyses were performed on an Agilent 8890N spectrometer equipped with a 30 m x 250 µm x 0.25 µm Agilent HP-5MS UI capillary column and operated under the same conditions. Products were characterized by comparison with the given literature, when possible.

Solid infrared spectra were recorded on an attenuated total reflection (ATR) Fourier transform infrared (FT-IR) spectroscopy, performed in a JASCO FT/IR–4700, which was employed to record the IR spectra from 400 to 4000 cm^{-1} of the different solid catalysts.

The cation content of the solids was determined by the inductively coupled plasma-atomic emission spectroscopy (ICP–AES) by disaggregation of the solid in aqueous acid mixture and filtration.

N_2 adsorption-desorption isotherms were performed at 77 K on sieved zeolites after outgassing for 16 h under vacuum.

X-ray diffraction (XRD) measurements were recorded in a CubiX PRO (PAN Analytical) spectrometer, with a Cu K(α) radiation source, 1.5406 Å wavelength.

Solid-state nuclear magnetic resonance spectra were recorded at room temperature with a Bruker AVIII HD 400 WB spectrometer. The ^{27}Al spectra were recorded with π/12 pulse length of 1 µs, and a recycle delay of 3 s, pinning the samples at 20 kHz.

FTIR-pyridine studies were performed in a Nicolet Is-10 Thermo FT-infrared spectrophotometer with self-supported pellets, degassed under vacuum at 300 ºC for 12 hours, followed by the introduction of pyridine into the cell at 650 Pa. After equilibrium

was attained, the cell was degassed at a desired experiment temperature and cooled down to room temperature. We then acquired FT-IR at 150 ºC (weak strength), 250 ºC (medium strength), and 350 ºC (high strength). A spectrum was collected under vacuum before pyridine adsorption, to be used as a background. We subtracted the background from each spectrum and normalized the absorbance to weight before calculations.

Field emission scanning electron microscopy (FESEM) were carried out after supporting the zeolite on a grid and measuring with a ZEISS Ultra-55 instrument, from Oxford Instruments. Samples were prepared by dropping the suspension of the solid sample in DCM directly onto holey-carbon-coated copper grids. For dealuminated H-Y zeolite, powder sample were embedded in an epoxy resin and sliced at a thickness of less than 100 nm with an ultramicrotome. They were then deposited on holey carbon copper grid before HR-TEM and EDX observation.

S1.3 Reaction procedures:

General procedure of the carbonyl-olefin/alkyne intramolecular metathesis reaction:

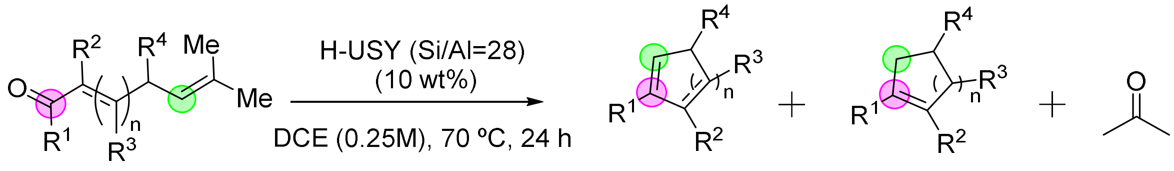

1 n= 1,2 **2** **2'** **3**

In a 6-7 mL vial equipped with a magnetic stirrer, 0.5 mmol of **1** was introduced and dissolved in DCE (0.25 M). Then, the corresponding amount of zeolite H-USY (Si/Al=28, 10 wt% respect to the total mixture) was added to the reaction mixture and left stirring for 24 h at a temperature of 70 °C, following the evolution of the reaction by GC and GC-MS. Samples were taken following the procedure described below: 25 µL of the reaction mixture was placed in a 2 mL chromatography vial containing CH_2Cl_2:*n*-dodecane (1000:1) solution (1 mL) as an external standard.

General procedure of the intermolecular carbonyl-olefin metathesis reaction:

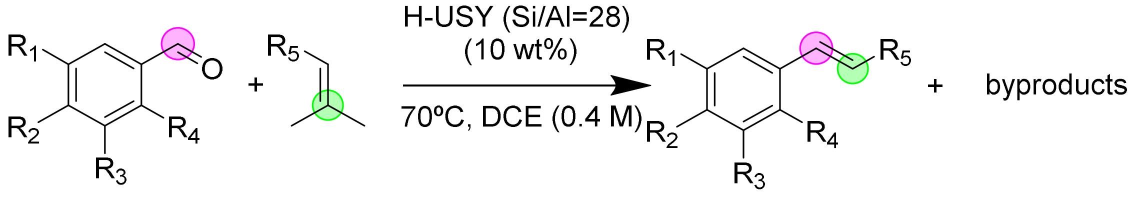

4 (0.2 mmol) **5** (2 equiv.) **6**

In a 6-7 mL vial equipped with a magnetic stirrer, 0.25 mmol of **4** and 2.5 equivalents of **5** were introduced dissolved in DCE when necessary (until 0.4 M). Then, the corresponding amount of H-USY (Si/Al=28) zeolite (50 mg, 10 wt%) was added to the reaction mixture and left stirring for 24 h at a temperature of 70 °C, following the evolution of the reaction by GC and GC-MS. Samples were taken following the procedure described below: 25 µL of the reaction mixture was placed in a 2 mL chromatography vial containing CH_2Cl_2:*n*-dodecane (1000:1) solution (1 mL) as an external standard.

General procedure of the carbonyl-alkyne intramolecular metathesis reaction:

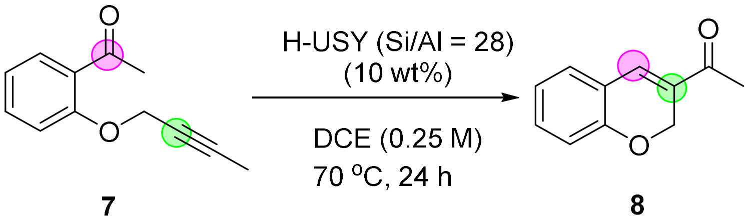

7 **8**

In a 6-7 mL vial equipped with a magnetic stirrer, 0.5 mmol of **7** was introduced and dissolved in DCE (0.25 M). Then, the corresponding amount of zeolite H-USY (Si/Al=28, 10 wt%) was added to the reaction mixture and left stirring for 24 h at a temperature of 70 °C, following the evolution of the reaction by GC and GC-MS. Samples were taken following the procedure described below: 25 µL of the reaction mixture was placed in a 2 mL chromatography vial containing CH_2Cl_2:*n*-dodecane (1000:1) solution (1 mL) as an external standard.

General procedure of the intermolecular carbonyl-alkyne metathesis reaction:

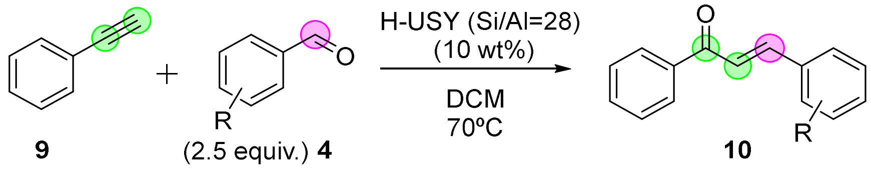

9 (2.5 equiv.) **4** **10**

In a 6-7 mL vial equipped with a magnetic stirrer, 0.25 mmol of **9** and 2.5 equivalents of **4** were introduced and both were dissolved in DCE (0.4 M) (in case of

using MeOH or absence of solvent will be indicated in the text). Then, the corresponding amount of H-USY (Si/Al=28) zeolite (30 mg) was added to the reaction mixture and left stirring for 24 h at a temperature of 70 °C, following the evolution of the reaction by GC and GC-MS. Samples were taken following the procedure described below: 25 µL of the reaction mixture was placed in a 2 mL chromatography vial containing CH_2Cl_2:*n*-dodecane (1000:1) solution (1 mL) as an external standard.

Hot filtration test: Following the general reaction procedure, two parallel reactions were carried out and one of them was rapidly filtered at the reaction temperature (70 ºC) after 20 min reaction time (~30% conversion). Then, the kinetic profiles for both the solid-containing reaction and the filtrates were assessed and compared. In this way, reagent **1a** was mixed in a sealed vial with a magnetic stirrer, H-USY zeolite (Si/Al=28, 10 wt%) and DCM (0.25M) and allowed to react at 70 °C. Once the reaction reached 30% conversion, the catalyst was filtered using a 0.22 µm syringe filter and the reaction was continued under the same conditions. A sample was taken after 24 h following the procedure explained above to see if there was a migration of active species into solution.

Zeolite H-USY (Si/Al=28) reuse test: Starting reagent **1a** (0.5 mmol) was mixed in a sealed vial equipped with a magnetic stirrer with H-USY zeolite (Si/Al=28, 10 wt%) and DCM (0.25M) and allowed to react at 70 °C for 24 h. After completion of the first catalytic cycle, the catalyst was washed with DCM four times centrifuging between washes to separate the liquid phase from the solid, dried under vacuum, calcined at 600 °C and reused again.

Representative reaction procedure for the H-USY (Si/Al=28) catalyzed reaction in flow: Starting reagent **1a** (16 mmol) was dissolved in DCM (0.25 M) in a beaker and placed in a 20.0 mL syringe. The mixture was pumped at atmospheric pressure at a flow rate of 0.1 $mL \cdot min^{-1}$ over a 10 mm inner diameter stainless steel tube filled with H-USY (Si/Al=28, 2 g), and SiC and glass wool to promote uniform flow distribution through the fixed bed and avoid preferential pathways. The reaction took place at room temperature and samples were collected by gravity. The collected samples were analyzed by GC using *n*-dodecane as an external standard.

S1.4 Synthesis of the starting materials:

Synthesis of starting materials for intramolecular COM reactions

Synthesis of 2-(2-methylprop-1-en-1-yl)benzaldehyde (S1): $Pd(PPh_3)_4$ (1 mol%, 115.5 mg) and Na_2CO_3 (3 equiv, 3.18 g) were added to an oven-dried round bottom flask equipped with a stir bar and a reflux condenser. The flask was sealed with a septum and 3 vacuum-nitrogen cycles were applied to obtain the presence of an inert atmosphere. Then, toluene (35 mL), EtOH (10 mL), distilled water (5 mL), 2-bromobenzaldehyde (10 mmol, 1.17 mL) and 2,2-dimethylethylenylboronic acid pinacolic ester (1.2 equiv) were added to the flask with a syringe. The reaction was refluxed to completion and, after cooling to room temperature, the mixture was diluted by adding water (50 mL) and extracted with Et_2O (3×50 mL). The organic phase was washed with brine (50 mL), dried over anhydrous Na_2SO_4 and concentrated at a rotary evaporator. The crude reaction product was purified by column chromatography (silica gel, hexanes/DCM =4/1 to 1/1) to afford the desired compound (82%) as a pale yellow oil.

Procedure 1: To an oven-dried round bottom flask equipped with a magnetic stirrer and a reflux condenser was added 1,3-dicarbonyl (2 mmol), K_2CO_3 (1.5 eq.), KI (0.75 eq.), anhydrous DMF (10 mL) and alkyl bromide (1.1 eq.). The reaction mixture was magnetically stirred at 55 °C under nitrogen atmosphere overnight. After completion of the reaction, it was stopped by addition of water (20 mL), extracted with Et_2O (3×10 mL), washed with brine, dried over Na_2SO_4 and finally concentrated at a rotary evaporator. The residue was purified by column chromatography (silica gel, *n*-hexane:ethyl acetate = 98/2 to 90/10) to give the desired products.

Procedure 2: In an oven-dried round bottom flask equipped with a magnetic stirrer and a reflux condenser, deoxybenzoin (2 mmol), *t*BuOK (1.5 eq.), anhydrous THF (10 mL) and alkyl bromide (1.5 eq.) were added. The reaction mixture was refluxed under nitrogen atmosphere overnight and after completion of the reaction, the mixture was stopped with water (20 mL), extracted with Et_2O (3×10 mL), washed with brine, dried using Na_2SO_4 and concentrated at a rotary evaporator. The final mixture obtained was purified by column chromatography (silica gel, *n*-hexane:ethyl acetate = 95/5) to obtain the desired products.

Procedure 3: Toluene (5 mL), AcOH (10 mol%), piperidine (10 mol%), diketone (2 mmol) and **S1** (2 mmol) were added to an oven-dried round-bottomed flask equipped with a magnetic stirrer and reflux. The resulting solution was refluxed until the reaction was complete. After cooling to room temperature, the reaction was stopped by adding distilled water (10 mL) and extracted with Et_2O (3×10 mL). The organic phase was

washed with brine (10 mL), dried using anhydrous Na_2SO_4 and concentrated at a rotary evaporator. The crude product was purified by column chromatography (silica gel, *n*-hexane:ethyl acetate = 9/1 to 4/1) to give the desired pure product.

Procedure 4: Magnesium turnings (6 mmol) and some iodine were added to an oven-dried round bottom flask equipped with a magnetic stirrer and a reflux condenser. The flask was sealed with a septum and then 3 vacuum-nitrogen cycles were applied. A 1 mL solution of aryl bromide (6.6 mmol) in dry THF (12 mL) was added by syringe. The reaction was started with a heat gun and then the rest of the halide solution was added little by little over 30 min. The mixture was stirred at room temperature until the magnesium was completely dissolved. After completion of the Grignard reaction citronellal (5 mmol) was slowly added and the resulting mixture was stirred at room temperature until the reaction was complete. After completion it was stopped using a saturated solution of NH_4Cl (20 mL), extracted with Et_2O (3 × 20 mL). The organic phase was washed with brine, dried over anhydrous Na_2SO_4 and concentrated at rotary evaporator to obtain the crude alcohol which was used directly without further purification. In a second reaction step, dried DCM (50 mL) was added to an oven-dried round-bottom flask equipped with a magnetic stirrer and cooled to -78 °C. Oxalyl chloride (2 M in DCM, 3 mL) and DMSO (5.5 mmol) were then added and the reaction was stirred for 20 min. Alcohol solution in DCM (5 mL) was then added and the reaction was stirred again for 20 minutes, after which triethylamine (25 mmol) was added. The resulting mixture was left at room temperature and stirred overnight. Subsequently, the reaction was stopped with a saturated NH_4Cl solution (20 mL), extracted with DCM (3× 20 mL). The organic phase was washed with brine, dried using anhydrous Na_2SO_4 and concentrated by rotary evaporation. The crude product was purified by column chromatography (silica gel, *n*-hexane:ethyl acetate = 98/2 to 90/10) to give the desired product.

S1.5 Synthesis of the oxetane intermediate and reactivity

Synthesis of Int-1

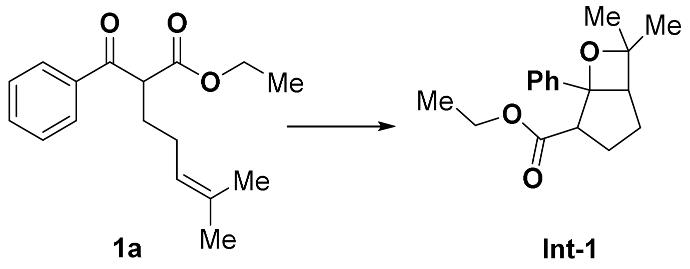

The corresponding oxetane was prepared via the Paterno-Büchi photocycloaddition of **1a**. Briefly, an acetonitrile solution (5 mL) of the **1a** (300 mg, 1.1 mmol) was placed in a Quartz tube and irradiated for 60 h under argon atmosphere with a multilamp photoreactor, using 8 W lamps (10x) with maximum emission at 350 nm. After irradiation, the solvent was evaporated, and the corresponding oxetane was isolated from the reaction mixture by means of high-performance liquid chromatographic (HPLC) using acetonitrile/water (90/10 v/v) as eluent. The intermediate obtained is the oxetane **Int-1.**

Synthesis of product 2a by the intermediate Int-1

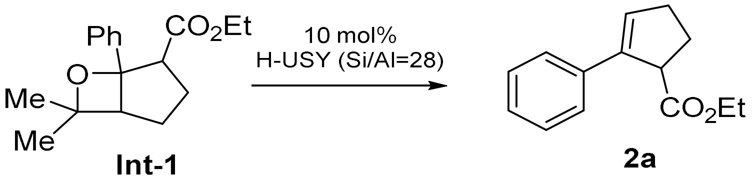

Int-1 was put in reaction conditions to observe if we were able to obtain the corresponding product **2a. Int-1** was mixed with zeolite H-USY (Si/Al=28, 10 mol%) and dissolved in DCE (0.25M). The reaction mixture was stirred for 15 minutes at 70 ºC, and a sample was taken following the procedure described for the general reaction: 25 μL of the reaction mixture were placed in a 2 mL chromatography vial containing CH_2Cl_2:*n*-dodecane (1000:1) solution (1 mL) as an external standard, and analysed by GC and GC-MS.

S1.6 Compound characterization.

The characterization of the different compounds obtained was carried out by GC-MS, ^{1}H NMR, ^{13}C NMR and DEPT techniques. In case of specific syntheses of any of the materials used as starting reagents, it will be indicated in the text together with their corresponding characterization.

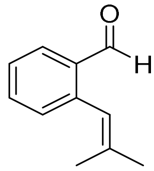

2-(2-Methylprop-1-en-1-yl)benzaldehyde (S1). GC-MS (m/z, M^+ 160): 160, 145 (100), 128, 115, 103, 91, 77. ^{1}H NMR (300 MHz, $CDCl_3$) δ 10.19 (d, *J* = 0.8 Hz, 1H), 7.86 (dd, *J* = 7.8, 1.5 Hz, 1H), 7.50 (td, *J* = 7.8, 1.5 Hz, 1H), 7.38 – 7.28 (m, 1H), 7.25 – 7.14 (m, 1H), 1.93 (d, *J* = 1.4 Hz, 3H), 1.63 (d, *J* = 1.4 Hz, 3H). ^{13}C NMR (75 MHz, $CDCl_3$) δ 192.7, 142.2, 139.2, 133.8, 133.6, 130.8, 128.1, 126.8, 121.3, 26.1, 19.4.

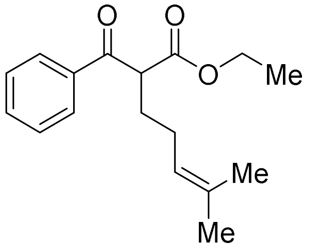

Ethyl 2-benzoyl-6-metilhept-5-enoate (1a). It was prepared according to the general synthesis procedure 1 from ethyl benzoylacetate and 5-bromo-2-methylpent-2-ene on a scale of 10 mmol to give compound **1a** as a yellowish oil.

GC-MS (m/z, M^+ 274): 274 (100), 201, 197, 191, 169, 105, 83, 77, 73, 55, 42. ^{1}H NMR (400 MHz, $CDCl_3$) δ 8.05 – 7.94 (m, 2H), 7.65 – 7.55 (m, 1H), 7.54 – 7.44 (m, 2H), 5.18–5.09 (m, 1H), 4.37 – 4.29 (m, 1H), 4.17 (qd, *J* = 7.1, 0.7 Hz, 2H), 2.14 – 2.02 (m, 4H), 1.69 (d, *J* = 1.4 Hz, 3H), 1.54 (d, *J* = 1.4 Hz, 3H), 1.20 (t, *J* = 7.1 Hz, 3H). ^{13}C NMR (101 MHz, $CDCl_3$) δ 195.4, 170.1, 136.3, 133.4, 128.7, 128.6, 123.0, 61.3, 53.4, 29.0, 25.9, 25.7, 17.6, 14.0.

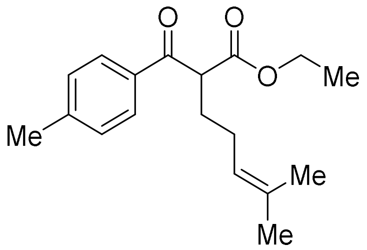

Ethyl 6-methyl-2-(4-methylbenzoyl)hept-5-enoate (1b). It was prepared according to general synthesis procedure 1 from ethyl 3-oxo-3-(*p*-tolyl)propanoate and 5-bromo-2-methylpent-2-ene on a 10 mmol scale to give compound **1b** as a yellowish oil.

GC-MS (m/z, M^+ 288): 288 (100), 243, 233, 215, 205, 197, 91, 83, 73, 55 45.

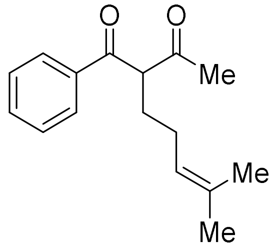

2-(4-Methylpent-3-en-1-yl)-1-phenylbutane-1,3-dione (1c). It was prepared according to general synthesis procedure 1 from 1-phenylbutane-1,3-dione and 5-bromo-2-methylpent-2-ene on a 10 mmol scale to give compound **1c** as a yellowish oil.

GC-MS (m/z, M^+ 244): 244, 229, 215, 201, 186, 175 (100), 163, 147, 131, 124, 115, 105, 91, 82, 77, 67, 55. ^{1}H NMR (401 MHz, $CDCl_3$) δ 7.97 (d, *J* = 7.0 Hz, 2H), 7.62 – 7.55 (m, 1H), 7.51 – 7.42 (m, 2H), 5.11 – 5.02 (m, 1H), 4.44 (t, *J* = 7.0 Hz, 1H), 2.13 (s, 3H), 2.06 – 1.92 (m, 4H), 1.64 (d, *J* = 1.4 Hz, 3H), 1.48 (d, *J* = 1.4 Hz, 3H). ^{13}C NMR (101 MHz, $CDCl_3$) δ 204.6, 196.7, 136.7, 133.7, 133.5, 128.9, 128.8, 123.1, 62.8, 29.3, 28.0, 26.1, 25.8, 17.8.

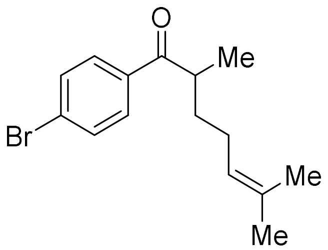

1-(4-Bromophenyl)-2,6-dimethylhept-5-en-1-one (1d). It was prepared according to general synthesis procedure 1 from 1-(4-bromophenyl)propan-1-one and 5-bromo-2-methylpent-2-ene on a 10-mmol scale to give compound **1d** as a yellowish oil.

GC-MS (m/z, M^+ 294): 294 (100), 279, 210, 182, 154, 139, 111, 83, 69, 55, 42, 15. ^{1}H NMR (401 MHz, $CDCl_3$) δ 7.80 (d, *J* = 8.6 Hz, 2H), 7.60 (d, *J* = 8.6 Hz, 2H), 5.16 – 4.96 (m, 1H), 3.40 (h, *J* = 6.8 Hz, 1H), 2.07 – 1.94 (m, 2H), 1.93 – 1.73 (m, 1H), 1.65 (d, *J* = 1.3 Hz, 3H), 1.51 (d, *J* = 1.4 Hz, 3H), 1.49 – 1.36 (m, 1H), 1.18 (d, *J* = 6.8 Hz, 3H). ^{13}C NMR (101 MHz, $CDCl_3$) δ 203.5, 135.6, 132.6, 132.0, 130.0, 128.0, 123.9, 40.1, 33.8, 25.9, 25.8, 17.8, 17.3.

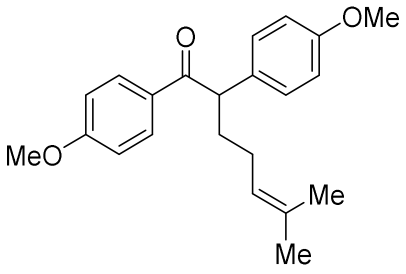

1,2-Bis(4-metoxyphenyl)-6-methylhept-5-en-1-one (1e). It was prepared according to general synthesis procedure 2 from 1,2-bis(4-methoxyphenyl)ethan-1-one and 5-bromo-2-methylpent-2-ene on a 10 mmol scale to give compound **1e** as a yellowish oil.

GC-MS (m/z, M^+ 256): 256, 241, 227, 212, 203, 187, 159, 148, 135 (100), 121, 107, 92, 77, 69. ^{1}H NMR (401 MHz, $CDCl_3$) δ 8.04 – 7.85 (m, 2H), 7.21 (d, *J* = 8.7 Hz, 2H), 6.86 (d, *J* = 8.7 Hz, 2H), 6.81 (d, *J* = 8.7 Hz, 2H), 5.21 – 4.95 (m, 1H), 4.47 (t, *J* = 7.2 Hz, 1H), 3.82 (s, 3H), 3.75 (s, 3H), 2.50 – 2.08 (m, 1H), 1.95 (q, *J* = 7.4 Hz, 2H), 1.88 – 1.72 (m, 1H), 1.66 (d, *J* = 1.4 Hz, 3H), 1.48 (d, *J* = 1.4 Hz, 3H). ^{13}C NMR (101 MHz, $CDCl_3$) δ 199.0, 163.3, 158.6, 132.4, 132.3, 131.0, 130.1, 129.4, 124.1, 114.3, 113.8, 55.5, 55.3, 51.6, 34.2, 26.1, 25.9, 17.9.

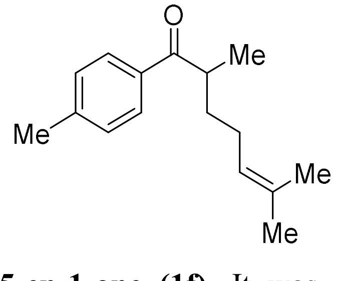

2,6-Dimethyl-1-(*p*-tolyl)hept-5-en-1-one (1f). It was prepared according to general synthesis procedure 1 from 1-(*p*-tolyl)propan-1-one and 5-bromo-2-methylpent-2-ene on a 10 mmol scale to give compound **1f** as a yellowish oil.

GC-MS (m/z, M^+ 230): 230, 216, 201, 188, 174, 161, 148 (100), 133, 119, 105, 91, 77, 65. ^{1}H NMR (401 MHz, $CDCl_3$) δ 7.91 – 7.70 (m, 2H), 7.27 – 6.94 (m, 2H), 5.05 – 4.98 (m, 1H), 3.38 (h, *J* = 6.8 Hz, 1H), 2.34 (s, 3H), 1.93 (q, *J* = 7.4 Hz, 2H), 1.85 – 1.73 (m,

1H), 1.59 (d, J = 1.3 Hz, 3H), 1.44 (d, J = 1.3 Hz, 3H), 1.44 – 1.31 (m, 1H), 1.11 (d, J = 6.9 Hz, 3H).

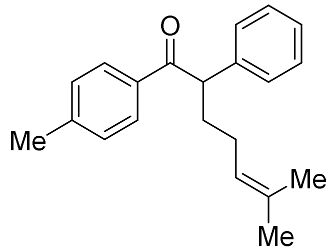

6-Methyl-2-phenyl-1-(*p*-tolyl)hept-5-en-1-one (1g). It was prepared according to general synthesis procedure 2 from 2-phenyl-1-(*p*-tolyl)ethan-1-one and 5-bromo-2-methylpent-2-ene on a 10 mmol scale to give compound **1g** as a yellowish oil.
GC-MS (m/z, M^+ 292): 292 (100), 215, 209, 201, 91, 83, 77, 69, 55, 42.

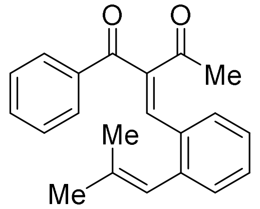

(*E*)-2-(2-(2-Methylprop-1-en-1-yl)benzilyden)-1-phenylbutane-1,3-dione (1h). It was prepared according to general synthesis procedure 3 from 1-phenylbutane-1,3-dione and 2-(2-methylprop-1-en-1-yl)benzaldehyde on a 10-mmol scale to give compound **1h** as a yellowish oil.
GC-MS (m/z, M^+ 304): 304, 286, 271, 261, 243, 228, 215, 207, 199, 182, 161, 157, 147, 142, 128, 115, 105 (100), 91, 77. ^{1}H NMR (401 MHz, $CDCl_3$) δ 7.99 (s, 1H), 7.87 – 7.78 (m, 1H), 7.54 – 7.43 (m, 1H), 7.42 – 7.30 (m, 3H), 7.23 – 7.14 (m, 2H), 7.13 – 7.08 (m, 1H), 6.96 (td, J = 7.5, 1.4 Hz, 1H), 6.33 (s, 1H), 2.38 (s, 3H), 1.98 (d, J = 1.4 Hz, 3H), 1.68 (d, J = 1.4 Hz, 3H). ^{13}C NMR (101 MHz, $CDCl_3$) δ 207.1, 198.0, 196.3, 141.1, 139.8, 138.7, 136.5, 133.9, 132.1, 130.3, 130.0, 129.3, 129.1, 128.9, 126.7, 122.9, 31.1, 26.3, 19.7.

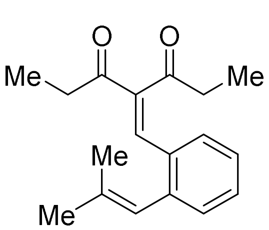

4-(2-(2-Methylprop-1-en-1-yl)benzilydene)heptane-3,5-dione (1i). It was prepared according to general synthesis procedure 3 from heptan-3,5-dione and 2-(2-methylprop-1-en-1-yl)benzaldehyde on a 10 mmol scale to give compound **1i** as a yellowish oil.
GC-MS (m/z, M^+ 270): 270, 252, 241, 223, 213, 199, 196, 183, 169, 165, 157, 142, 128, 115, 99, 91, 77, 57 (100). ^{1}H NMR (300 MHz, $CDCl_3$) δ 7.63 (s, 1H), 7.26 (ddd, *J* = 8.4, 6.7, 2.0 Hz, 1H), 7.19 – 7.06 (m, 3H), 6.23 – 6.17 (m, 1H), 2.63 (q, *J* = 7.2 Hz, 2H), 2.31 (q, *J* = 7.2 Hz, 2H), 1.88 (d, *J* = 1.5 Hz, 3H), 1.60 (d, *J* = 1.5 Hz, 3H). ^{13}C NMR (101 MHz, $CDCl_3$) δ 208.5, 199.3, 142.3, 139.4 138.7, 138.7, 132.4, 130.3, 129.9, 128.7, 126.8, 122.8, 37.5, 32.4, 26.3, 19.7, 8.3, 7.8.

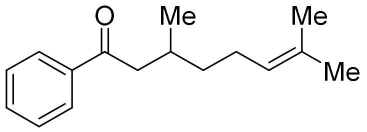

3,7-Dimethyl-1-phenyloct-6-en-1-one (1j). It was prepared according to the general synthesis procedure 4 from citronellal and bromobenzene on a 10 mmol scale to give compound **1j** as a yellowish oil.
GC-MS (m/z, M^+ 230): 230 (100), 188, 161, 125, 119, 111,105, 69, 42.

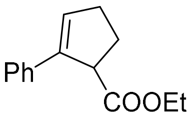

Ethyl 2-phenylciclopent-2-en-1-carboxilate (2a). GC-MS (m/z, M^+ 216): 216 (100), 189, 143, 139, 79, 73, 27. ^{1}H NMR (401 MHz, $CDCl_3$) δ: 7.36 – 7.27 (m, 2H), 7.27 – 7.20 (m, 2H), 7.17 (s, 1H), 5.96 (s, 1H), 4.24 – 4.08 (m, 2H), 3.11 (s, 1H), 2.45 – 2.07 (m, 4H), 1.41 – 1.26 (m, 3H). ^{13}C NMR (101 MHz, CDCl3) δ 175.5, 141.3, 135.6, 130.3, 128.5, 127.4, 126.0, 60.7, 51.5, 32.7, 29.5, 14.3.
^{1}H NMR (500 MHz, Chloroform) δ 7.36 – 7.27 (m, 2H), 7.27 – 7.20 (m, 2H), 7.17 (s, 1H), 5.96 (s, 1H), 4.24 – 4.08 (m, 2H), 3.11 (s, 1H), 2.45 – 2.07 (m, 4H), 1.41 – 1.26 (m, 3H).

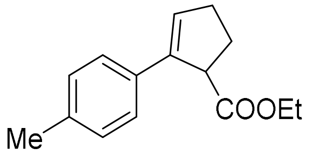

Ethyl 2-(*p*-tolyl)ciclopent-2-en-1- carboxilate (2b). GC-MS (m/z, M^+ 230): 230 (100), 215, 202, 157, 139, 91, 73, 28, 15.

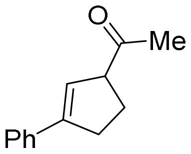

1-(3-Phenylciclopent-2-en-1-yl)etan-1-one (2c). GC-MS (m/z, M^+ 186): 186, 171, 158, 143 (100), 128, 115, 105, 91, 77.

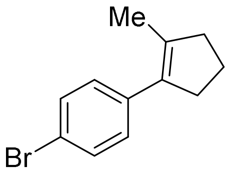

1-Bromo-4-(2-methyl-1-en-1-yl)benzene (2d). GC-MS (m/z, M^+ 236): 236 (100), 221, 157, 142 (100), 129, 115, 102, 91, 77,64. ^{1}H NMR (401 MHz, $CDCl_3$) δ 7.46 – 7.42 (m, 2H), 7.18 – 7.12 (m, 2H), 2.76 – 2.64 (m, 2H), 2.53 – 2.44 (m, 2H), 1.96 – 1.85 (m, 2H), 1.84 – 1.81 (m, 3H). ^{13}C NMR (101 MHz, $CDCl_3$) δ 137.8, 136.4, 131.2, 129.4, 119.8, 40.3, 37.3, 21.9, 15.6.

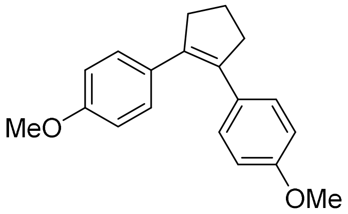

1,2-Bis(4-metoxyphenyl)ciclopent-1-ene (2e). GC-MS (m/z, M^+ 280): 280, 265, 249, 237, 221, 208, 202, 189, 172, 165, 159, 140, 132, 121, 115, 103, 91, 77. ^{1}H NMR (300 MHz, $CDCl_3$) δ 7.18 – 7.05 (m, 1H), 6.87 – 6.65 (m, 1H), 3.78 (s, 2H), 2.85 (t, *J* = 7.5 Hz, 1H), 2.08 – 1.95 (m, 1H). ^{13}C NMR (75 MHz, $CDCl_3$) δ 158.1, 135.7, 131.1, 129.3, 113.5, 55.2, 39.0, 21.9.

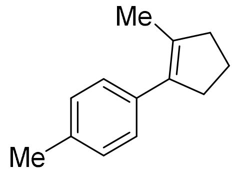

1-Methyl-4-(2-methylciclopent-1-en-1-yl)benzene (2f). GC-MS (m/z, M^+ 172): 172, 157, 142, 129, 115, 105, 91, 77. ^{1}H NMR (401 MHz, $CDCl_3$) δ 7.19 (d, *J* = 8.2 Hz, 2H), 7.14 (d, *J* = 7.8 Hz, 2H), 2.78 – 2.65 (m, 2H), 2.55 – 2.43 (m, 2H), 2.34 (s, 3H), 1.97 – 1.86 (m, 2H), 1.87 – 1.79 (m, 3H).

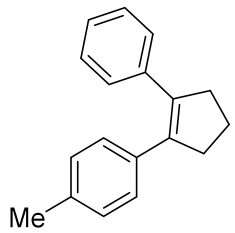

1-Methyl-4-(2-phenylciclopent-1-en-1-yl)benzene (2g). GC-MS (m/z, M^+ 234): 234 (100), 219, 157, 143, 91, 77, 15.

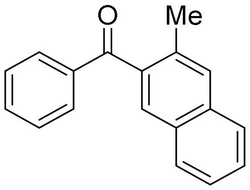

(3-Methylnaphtalen-2-yl)(phenyl)metanone (2h). GC-MS (m/z, M^+ 246): 246, 231, 215, 202, 189, 178, 169, 141, 126, 115, 105, 89, 77, 51. ^{1}H NMR (401 MHz, $CDCl_3$) δ 7.91 – 7.77 (m, 5H), 7.73 (d, *J* = 1.3 Hz, 1H), 7.66 – 7.58 (m, 1H), 7.58 – 7.51 (m, 1H), 7.51 – 7.43 (m, 3H), 2.50 (d, *J* = 1.0 Hz, 3H). ^{13}C NMR (101 MHz, $CDCl_3$) δ 198.5, 138.1, 137.4, 134.4, 133.9, 133.3, 130.9, 130.4, 129.3, 129.2, 128.6, 128.4, 127.7, 127.3, 126.1, 20.5.

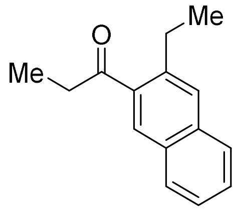

1-(3-Ethylnaphtalen-2-yl)propan-1-one (2i). GC-MS (m/z, M^+ 212): 212, 183 (100), 165, 153, 141, 128, 115, 101, 91, 77. ^{1}H NMR (401 MHz, $CDCl_3$) δ 8.08 (s, 1H), 7.85 (d, *J* = 8.1 Hz, 1H), 7.79 (d, *J* = 8.1 Hz, 1H), 7.69 (s, 1H), 7.59 – 7.51 (m, 1H), 7.51 – 7.43 (m, 1H), 3.06 (q, *J* = 7.5 Hz, 2H), 2.99 (q, *J* = 7.5 Hz, 2H), 1.27 (q, *J* = 7.5 Hz, 6H). ^{13}C

NMR (101 MHz, $CDCl_3$) δ 201.1, 140.4, 137.4, 134.7, 131.1, 128.8, 128.5, 128.4, 127.9, 127.3, 126.1, 35.4, 27.1, 16.0, 8.7.

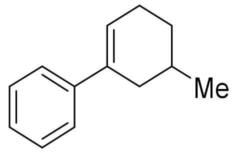

3-Methyl-2,3,4,5-tetrahydro-1,1'-biphenyl (2j). GC-MS (m/z, M^+ 172): 172 (100), 157, 116, 56, 95, 77, 15.

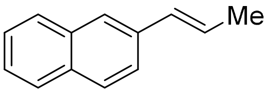

(*E*)-2-(Prop-1-en-1-yl)naphtalene (6aa). GC-MS (m/z, M^+160): 168, 140, 127, 92, 76, 41, 28. ^{1}H NMR (401 MHz, $CDCl_3$) δ 7.85-7.41 (m, 7H), 6.57 (dd, J = 15.6, 2.0 Hz, 1H), 6.36 (dq, J = 15.6, 6.4 Hz, 1H), 1.95 (d, J = 6.4, 1.6, 3H). ^{13}C NMR (101 MHz, $CDCl_3$) δ 135.5, 133.9, 131.6, 130.4, 128.9, 128.1, 126.8, 125.1, 124.5, 123.6, 121.9, 105.6, 18.8.

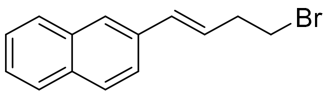

(*E*)-2-(4-Bromobut-1-en-1-yl)naphtalene (6ab). GC-MS (m/z, M^+ 260): 262-260 (100), 181, 167, 152, 141, 115, 89, 76.

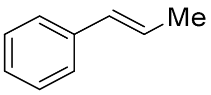

(*E*)-Prop-1-en-1-ilbenzene (6ba). GC-MS (m/z, M^+ 118): 118 (100), 90, 77, 41, 28.

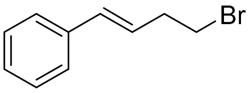

(*E*)-(4-Bromobut-1-en-1-yl)benzene (6bb). GC-MS (m/z, M^+ 210):212-210 (100), 131, 119, 106, 103, 90 78, 77.

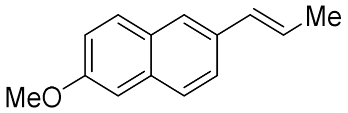

(*E*)-2-Metoxy-6-(prop-1-en-1-yl)naphtalene (6ca). GC-MS (m/z, M^+ 198): 198, 170, 167, 157, 41, 31, 28. ^{1}H NMR (300 MHz, $CDCl_3$) δ 7.74-7.53 (m, 4H), 7.44 – 7.34 (m, 2H), 6.55 (d, *J* = 15.0 Hz, 1H), 6.36 (dq, *J* = 15, 6.0 Hz, 1H), 3.93 (s, 3H), 1.95 (dd, *J* = 6.0, 1.5, 3H). ^{13}C NMR (75 MHz, $CDCl_3$) δ 157.4, 133.7, 133.3, 131.1, 129.3, 129.1, 126.9, 125.0, 124.9, 124.0, 118.8, 105.8, 55.2, 18.6.

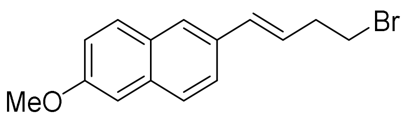

(*E*)-2-(4-bromobut-1-en-1-yl)-6-methoxynaphthalene (6cb). GC-MS (m/z, M^+ 198): 198, 170, 167, 157, 41, 31, 28.

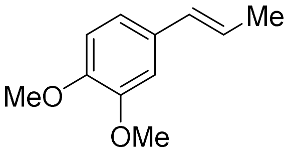

(*E*)-1,2-Dimetoxy-4-(prop-1-en-1-yl)benzene (6da). GC-MS (m/z, M^+ 178): 178 (100), 150, 147, 137, 116, 41, 31, 28. ^{1}H NMR (300 MHz, $CDCl_3$) δ 6.82-6.70 (m, 4H), 6.27 (dd, *J* = 15.6, 1.8, Hz, 1H), 6.02 (dq, *J* = 15.6, 6.3, Hz, 1H), 3.81 (s, 3H), 3.79 (s, 3H), 1.80 (dd, *J* = 6.3, 1.5, 3H). ^{13}C NMR (75 MHz, $CDCl_3$) δ 149.1, 148.2, 131.2, 130.7, 123.9, 118.7, 111.3, 108.6, 55.6, 55.8, 18.4.

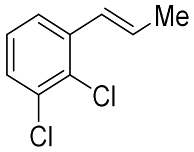

(*E*)-1,2-Dichloro-3-(prop-1-en-1-yl)benzene (6ea). GC-MS (m/z, M^+ 187): 187 (100), 158, 145, 116, 75, 41, 34, 28.

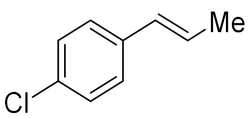

1-Chloro-4-(prop-1-en-1-yl)benzene (6fa). GC-MS (m/z, M^+ 152): 152 (100), 124,117, 111, 41, 35, 28. ^{1}H NMR (300 MHz, $CDCl_3$) δ 7.29 (dt, J = 8.7, 2.1 Hz, 2H), 7.23 (dt, *J* = 8.7, 2.1 Hz, 2H), 6.40 (*Z* dq, *J* = 11.5, 2.1 Hz, 1H), 6.37 (*E* dq, *J* = 15.6, 1.5 Hz, 1H), 6.24 (*E* dq, *J* = 15.6, 6.3 Hz, 1H), 5.84 (Z dq, *J* = 11.5, 7.2 Hz, 1H), 1.92 (dd, *J* =6.2 1.5

Hz, 3H). ^{13}C NMR (75 MHz, $CDCl_3$): δ 130.2, 130.0, 128.9, 128.7, 128.4, 127.6, 127.2, 126.6, 18.6.

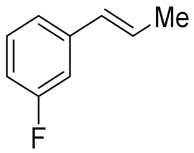

(*E*)-1-Fluoro-3-(prop-1-en-1-yl)benzene (6ga). GC-MS (m/z, M^+ 136): 136 (100), 117, 108, 95, 41, 28, 18.

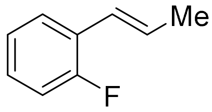

(*E*)-1-Fluoro-2-(prop-1-en-1-yl)benzene (6ha). GC-MS (m/z, M^+ 136): 136 (100), 117, 108, 95, 41, 28, 18.

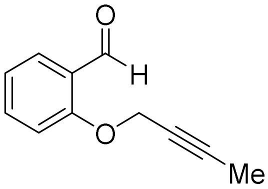

2-(But-2-in-1-iloxy)benzaldehyde (7). GC-MS (m/z, M^+ 174): 174, 159, 145, 131, 121 (100), 115, 103, 92, 77, 65, 53. ^{1}H NMR (401 MHz, $CDCl_3$) δ 10.48 (d, *J* = 0.8 Hz, 1H), 7.84 (dd, *J* = 7.7, 1.9 Hz, 1H), 7.63 – 7.40 (m, 1H), 7.11 (d, *J* = 8.4 Hz, 1H), 7.06 (t, *J* = 7.7 Hz, 1H), 4.78 (q, *J* = 2.3 Hz, 2H), 1.85 (t, *J* = 2.3 Hz, 3H). ^{13}C NMR (101 MHz, $CDCl_3$) δ 189.9, 160.3, 135.8, 128.5, 125.6, 121.4, 113.5, 84.9, 73.4, 57.2, 31.0.

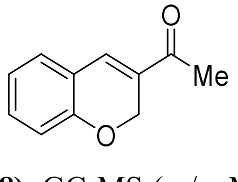

1-(2*H*-chromen-3-yl)etan-1-one (8). GC-MS (m/z, M^+ 174): 174, 159, 145, 131 (100), 115, 103, 87, 77, 51.

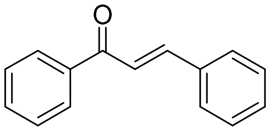

(*E*)-Chalcone (10b). GC-MS (m/z, M+ 208): 208 (100), 192, 131, 118, 90, 77, 15.

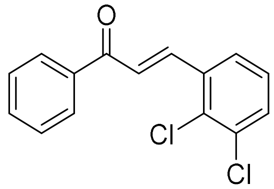

(*E*)-3-(2,3-Dichlorophenyl)-1-phenylprop-2-en-1-one (10d). GC-MS (m/z, M^+ 277): 277 (100), 241, 198, 165, 144, 139, 111, 77, 51, 16.

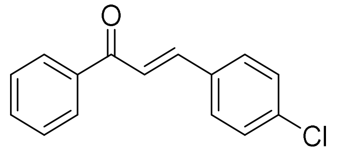

(*E*)-3-(4-Chlorophenyl)-1-phenylprop-2-en-1-one (10f). GC-MS (m/z, M^+ 243): 243 (100), 207, 165, 131, 124, 118, 111, 76, 15.

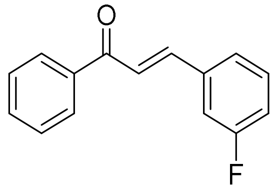

(*E*)-3-(3-Fluorophenyl)-1-phenylprop-2-en-1-one (10g). GC-MS (m/z, M^+226): 226 (100), 197, 105, 77, 51. ^{1}H NMR (401 MHz, $CDCl_3$) δ 8.02 (dm, J = 6.8, 2H), 7.77 (d, J = 15.6 Hz, 1H), 7.61 (tt, J = 6.0, 1.6 Hz, 1H), 7.55-7.51 (m, 3H), 7.42-7.33 (m, 4H), 7.14-7.10 (m, 1H). ^{13}C NMR (101 MHz, $CDCl_3$) δ 190.4, 163.2 (d, $J_{C\text{-}F}$ = 245.4 Hz), 143.4 (d, $J_{C\text{-}F}$ = 3.0 Hz), 138.1, 137.3 (d, $J_{C\text{-}F}$ = 8.1 Hz), 133.2, 130.7 (d, $J_{C\text{-}F}$ = 8.1 Hz), 128.7 (d, $J_{C\text{-}F}$ = 17.2 Hz), 124.7 (d, $J_{C\text{-}F}$ = 3.0 Hz), 123.4, 117.5 (d, $J_{C\text{-}F}$ = 22.0 Hz), 114.6 (d, $J_{C\text{-}F}$ = 22.0 Hz).

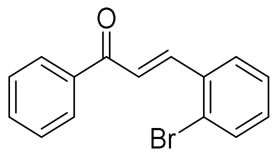

(*E*)-3-(2-Bromophenyl)-1-phenylprop-2-en-1-one (10i). GC-MS (m/z, M^+ 286): 287-285 (100), 208, 167, 154, 131, 118, 79, 77, 16. ^{1}H NMR (401 MHz, $CDCl_3$) δ 8.13 (d, J = 15.6, 1H), 8.02 (dm, J = 5.4, 2H), 7.74 (dd, J = 7.9, 1.6 Hz, 1H), 7.65 (dd, J = 7.9, 1.2 Hz, 1H), 7.6 (tt, J = 7.6, 1.6, 1H), 7.52 (t, J = 7.9, 2H), 7.43 (d, J = 15.6 Hz, 1H), 7.37 (t,

J = 7.6 Hz, 1H), 7.26 (m, 1H). ^{13}C NMR (101 MHz, $CDCl_3$) δ 190.6, 143.4, 138.1 135.3, 133.7, 133.1, 131.5, 128.8, 128.0, 127.9, 126.0, 125.3, 127.7, 127.6, 127.5.

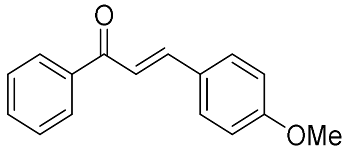

(*E*)-3-(4-Metoxyphenyl)-1-phenylprop-2-en-1-one (10j). GC-MS (m/z, M^+ 238): 238 (100), 161, 131, 120, 118, 107, 77, 16.

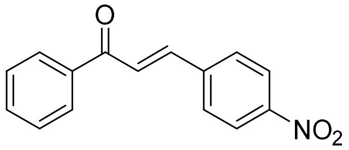

(*E*)-3-(4-Nitrophenyl)-1-phenylprop-2-en-1-one (10k). GC-MS (m/z, M^+ 253): 253 (100), 236, 176, 105, 77, 51.

S2. Characterization of the solid acids

S2.1 Summary of physicochemical parameters.

Table S1. Acidity of the different catalyst employed in this work.

Type of catalyst	Catalyst	Si/Al ratio	Acidity (mmol H^+/g)
Zeolitic	ZSM5		0.297
	MCM 41		0.087
	Z-β		0.204
	Z-HY (CBV 720)	15	0.274
	Z-HY (CBV 740)	20	0.131
	Z-HY (CBV 760)	28	0.087
	Z-HY (CBV 780)	40	0.087
	NaY-100		0
	Silicoalumina		0.02-0.1
	Acidic Alumina		0.02-0.1
Sulfonic	Amberlyst A-15		4.7
	Amberlyst A-16		3.3
	Amberlyst BD20		5.1
	Nafion SAC-13		0.3

S2.2 Brunauer, Emmett and Teller (BET) surface area.

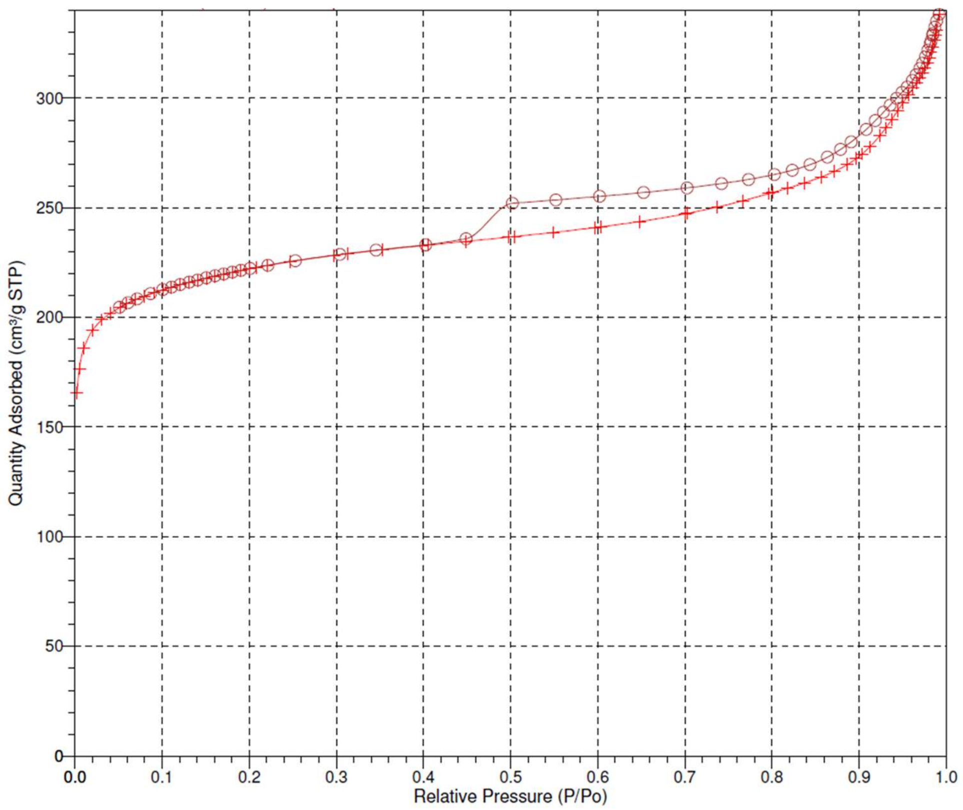

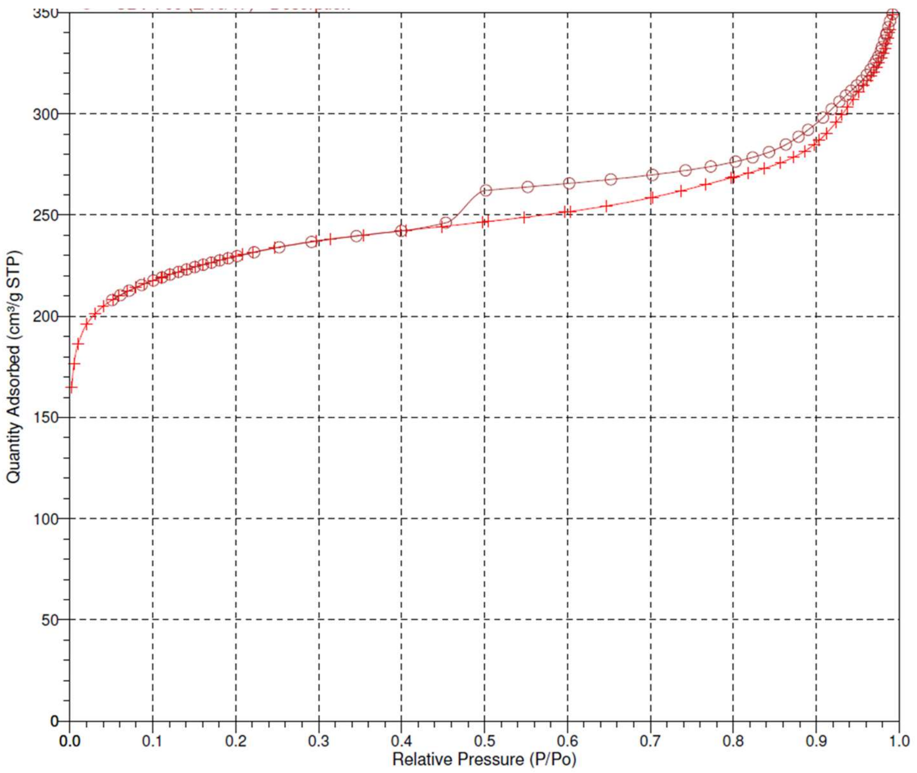

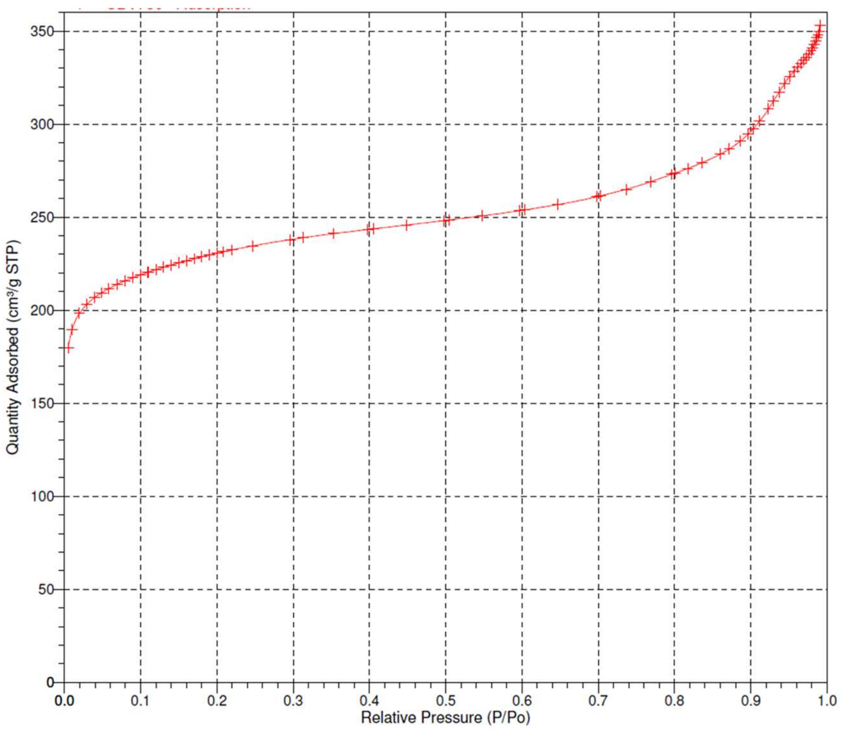

Figure S1. Isotherm plots for H-USY zeolites with Si/Al ratio of 15 (top), 30 (middle) and 40 (bottom). Red curve for adsorption data and brown curve for desorption curve. The corresponding BET surface areas are: 723.3817 ± 18.0572 m^2/g (Si/Al = 15), 753.3406 ± 17.7507 m^2/g (Si/Al = 28), 755.5590 ± 17.9760 m^2/g (Si/Al = 40).

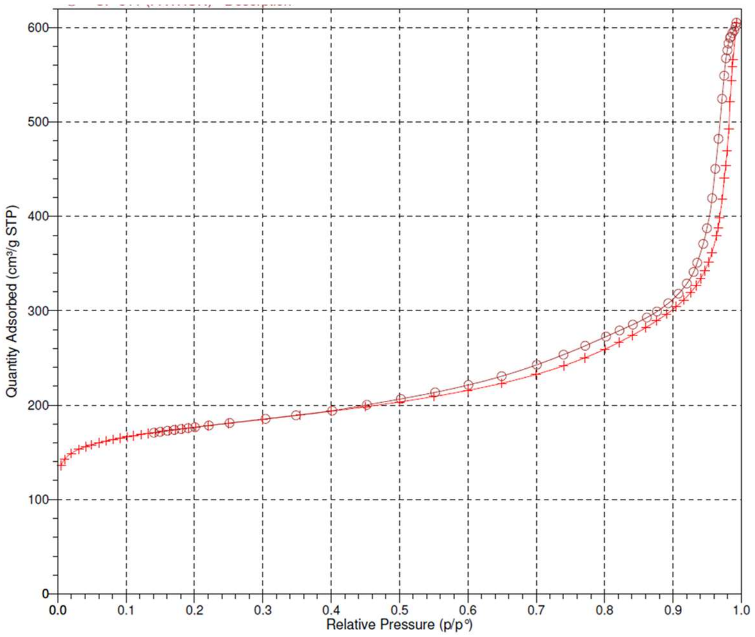

Figure S2. Isotherm plot of the H-Beta zeolite. Red curve for adsorption data and brown curve for desorption curve. The corresponding BET surface areas is 580.9233 ± 12.6809 m^2/g.

S2.3 Powder X-ray diffraction (PXRD).

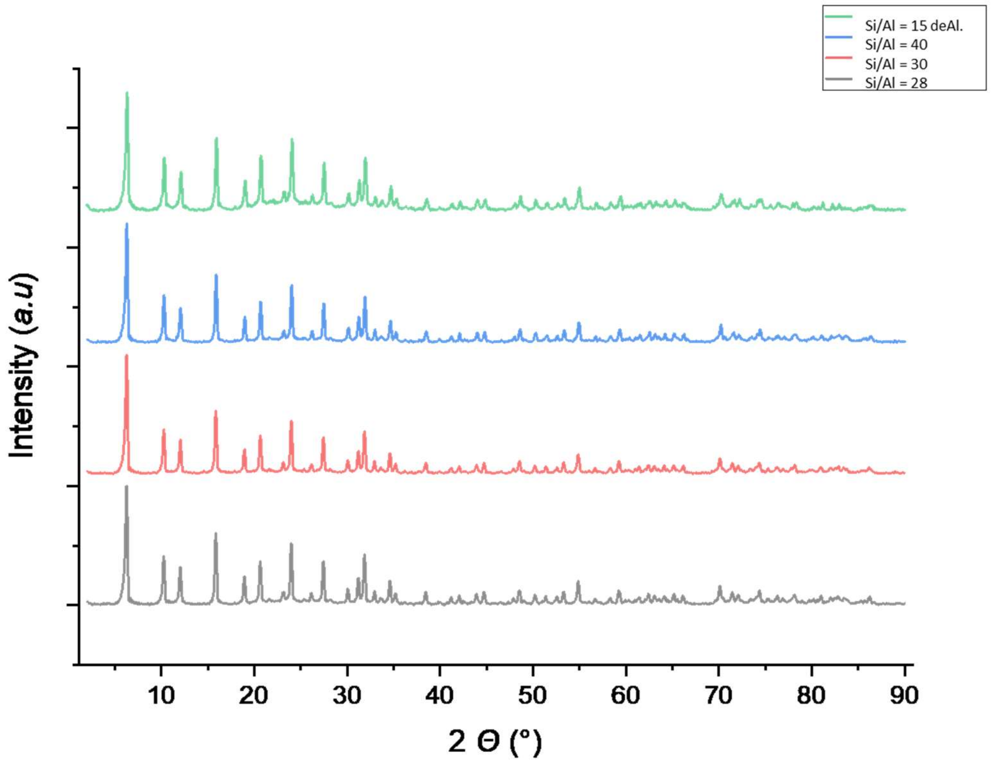

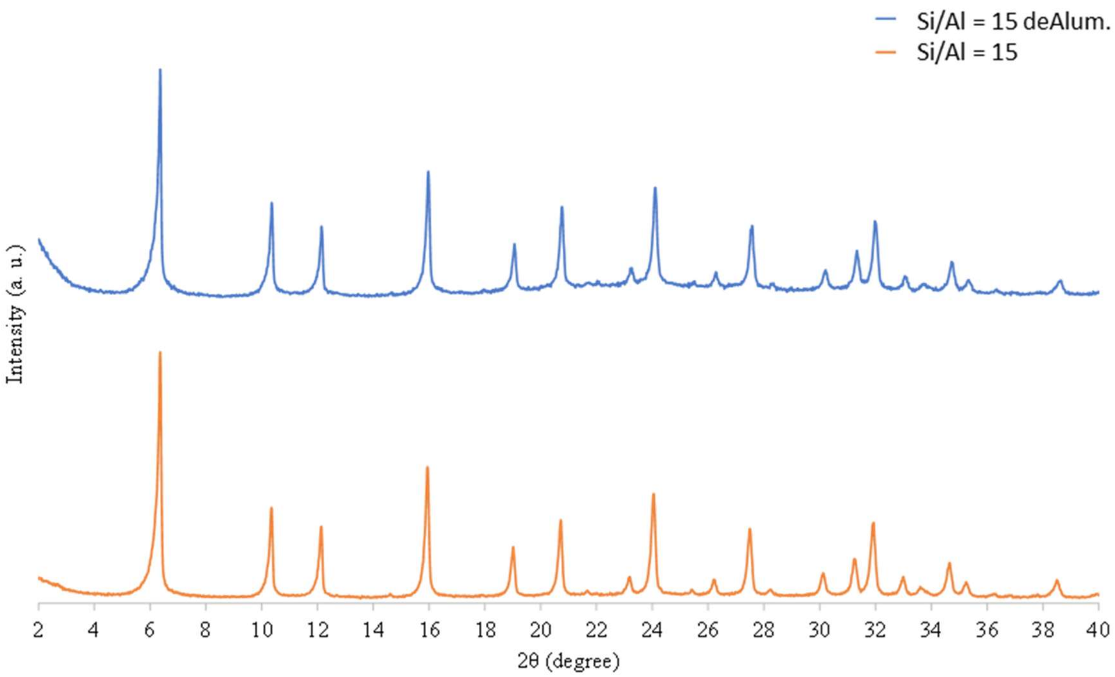

Figure S3. Comparative powder X-ray diffraction (PXRD) analysis of the different HUS-Y zeolites (top). Comparison of the zeolite (Si/Al =15) further dealuminated or not (bottom).

S2.4 FESEM and HRTEM.

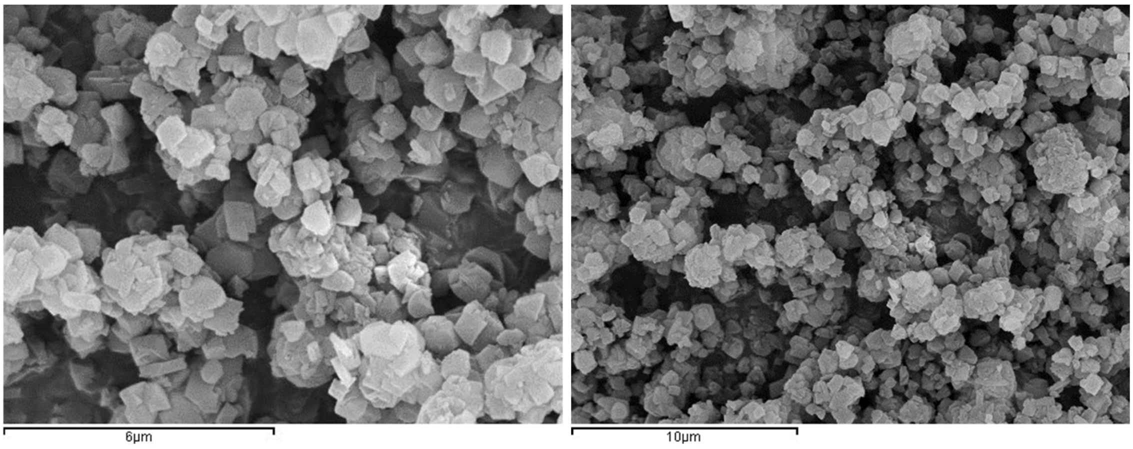

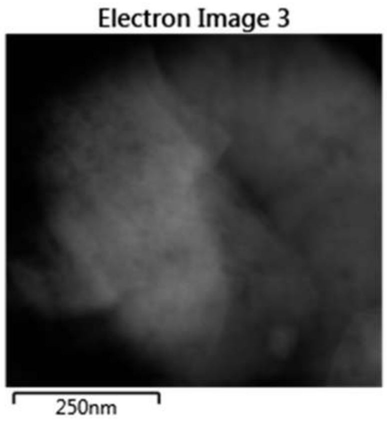

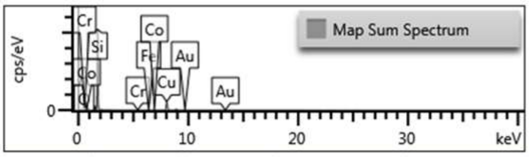

Element	Line Type	k Factor	k Factor type	Absorption Correction	Wt%	Wt% Sigma
C	K series	2.769	Theoretical	1	0.25	0.03
O	K series	2.02	Theoretical	1	36.06	0.12
Al	K series	1.049	Theoretical	1	2.19	0.04
Si	K series	1	Theoretical	1	47.68	0.12
Cr	K series	1.114	Theoretical	1	0.1	0.01
Fe	K series	1.143	Theoretical	1	0.09	0.01
Co	K series	1.185	Theoretical	1	0.12	0.02
Cu	K series	1.247	Theoretical	1	12.57	0.07
Au	L series	2.287	Theoretical	1	0.95	0.06
Total:					100	

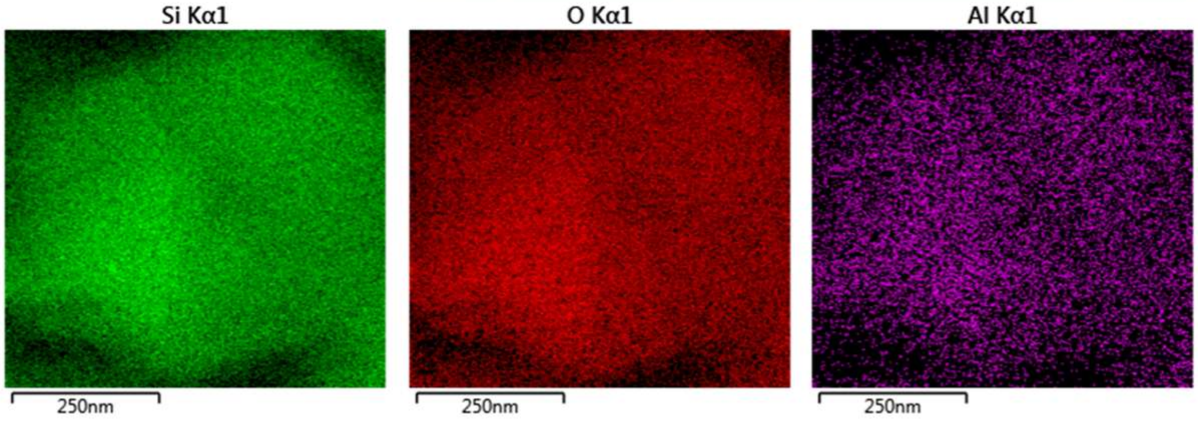

Figure S4. Representative field emission scanning electron microscope (FESEM) images of H-USY zeolite with Si/Al=15 (top) and the corresponding high-resolution transmission electron microscopy (HR-TEM) images, with the electron diffraction X-ray (EDX) analysis and mapping.

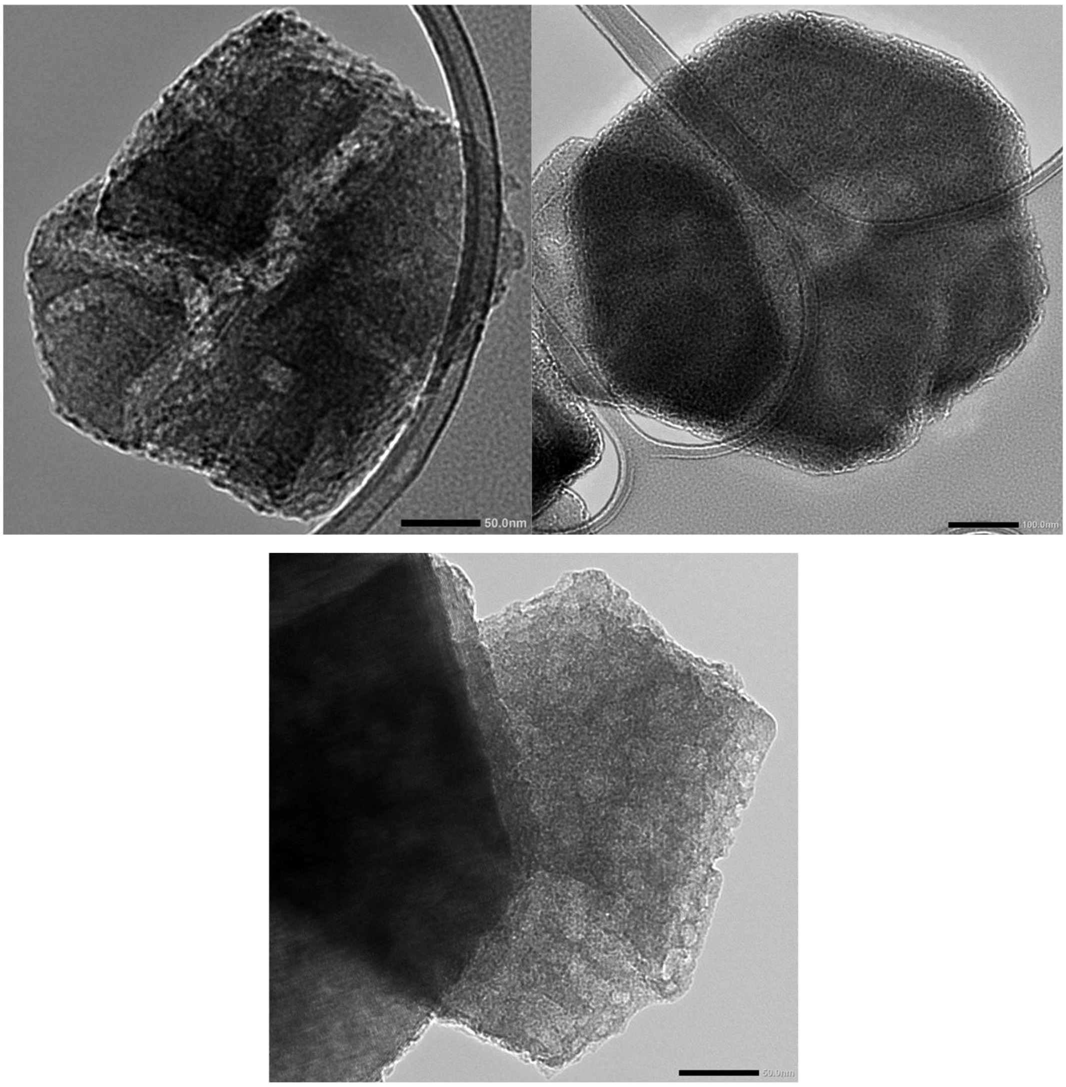

Figure S5. Different high-resolution transmission electron microscopy (HR-TEM) images, for 100 nm (left) and 50 nm (right) scale bars, of H-USY zeolite Si/Al=15 (top) and Si/Al=20 (bottom).

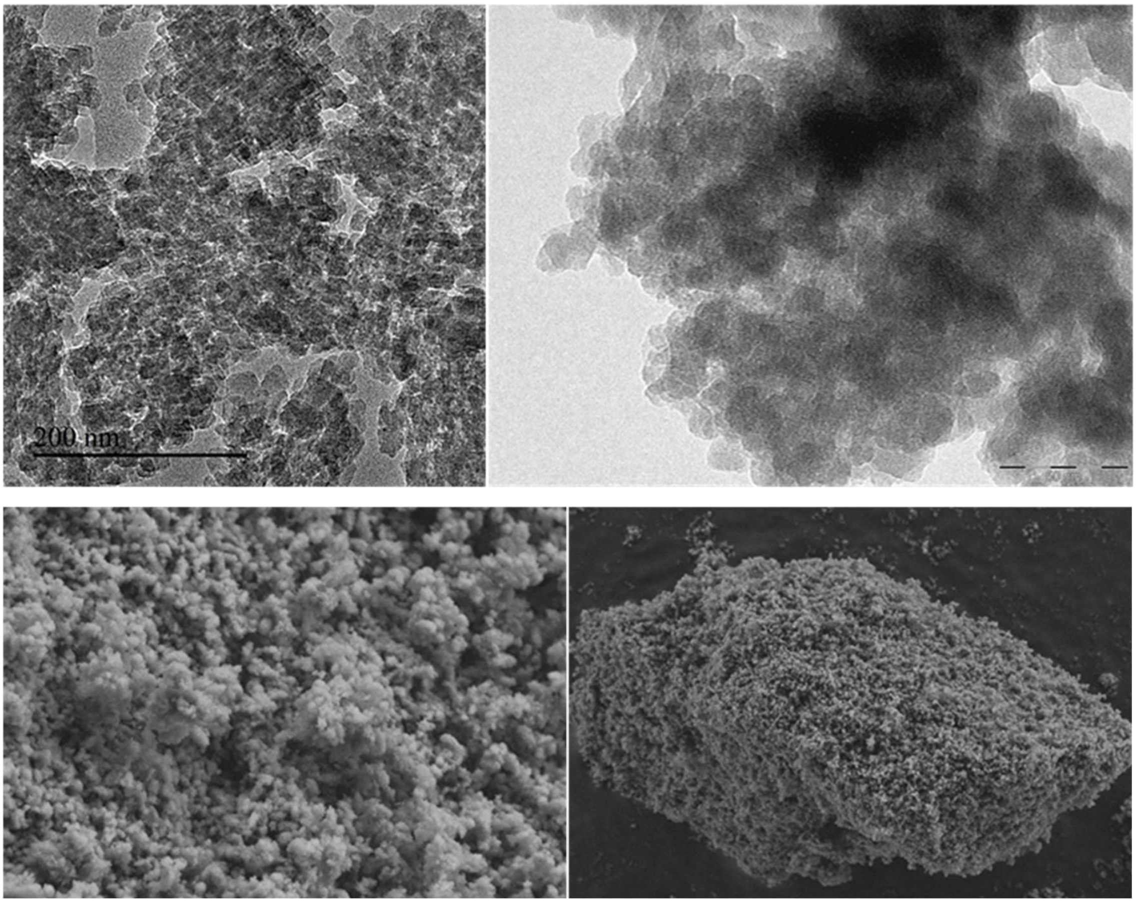

Figure S6. High-resolution transmission electron microscopy (HR-TEM, bottom) images of the H-Beta zeolite (top) and representative field emission scanning electron microscope (FESEM) images of the H-Beta zeolite (bottom).

S2.5 Acidity measurements.

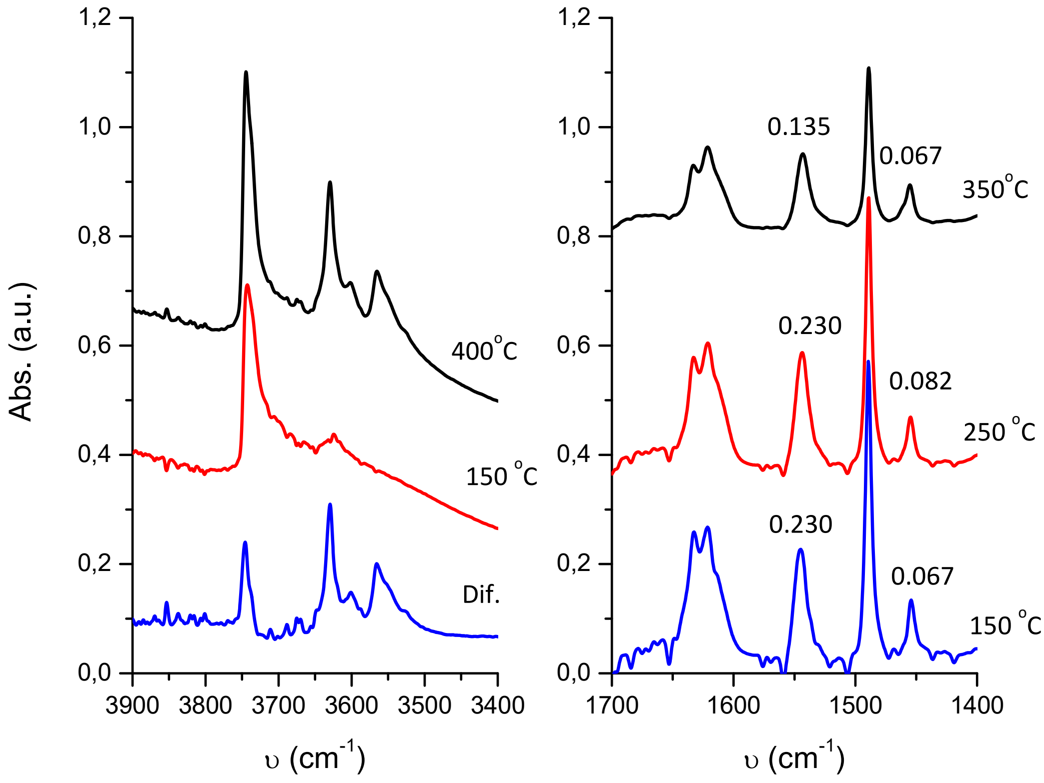

Figure S7. Pyridine titrations of the H-USY zeolite (Si/Al = 15), followed by in situ FT-IR after desorption under vacuum at different temperatures. Left: FT-IR spectra of H-Y zeolite at 150, 400 ºC and the difference between them. Right: FT-IR spectra of pyridine adsorbed HY zeolite at 150, 250 and 350 ºC. The three bands observed in the OH stretching region at 3746, 3630 and 3566 cm^{-1} are attributed to silanol groups, acidic OH groups in supercages (β cages, bridging hydroxyl) and acidic OH groups in sodalite cages (α cages, bridging hydroxyl), respectively. The band attributed to pyridine molecules coordinated to Lewis acid sites and that to pyridinium ions formed by protonation of pyridine on Brønsted acid sites are observed at 1455 and 1544 cm^{-1}, respectively.

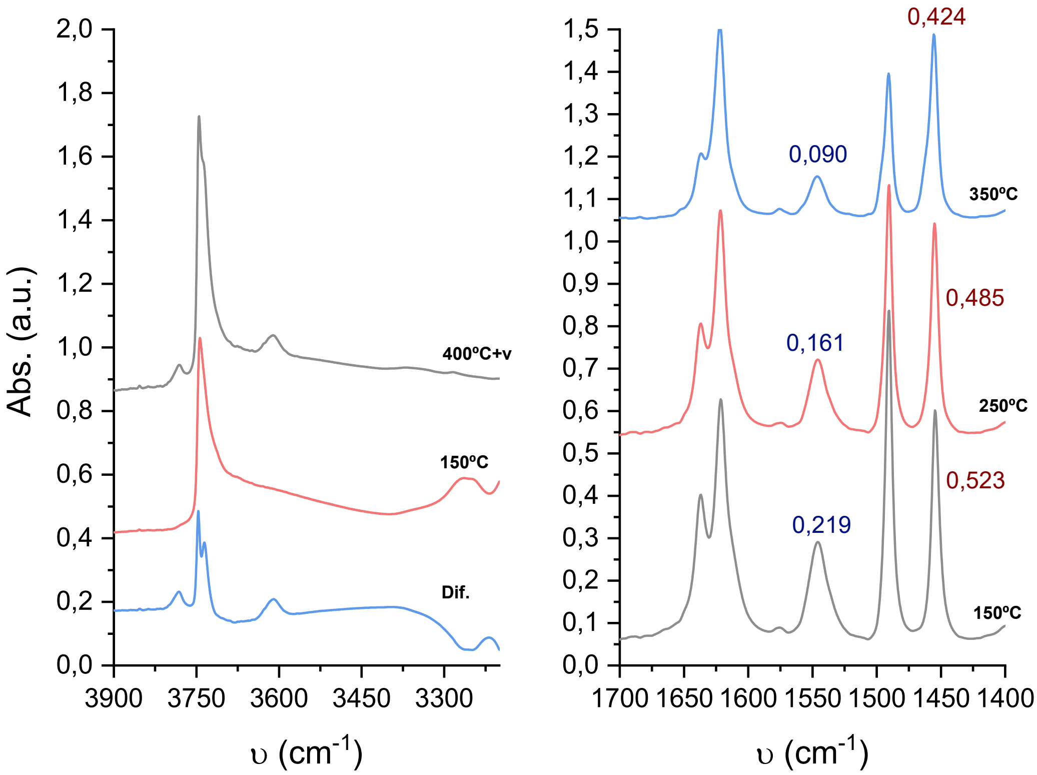

Figure S8. Pyridine titrations of the H-Beta zeolite, followed by in situ FT-IR after desorption under vacuum at different temperatures. Left: FT-IR spectra of H-Beta zeolite at 150, 400 ºC and the difference between them. Right: FT-IR spectra of pyridine adsorbed H-Beta zeolite at 150, 250 and 350 ºC.

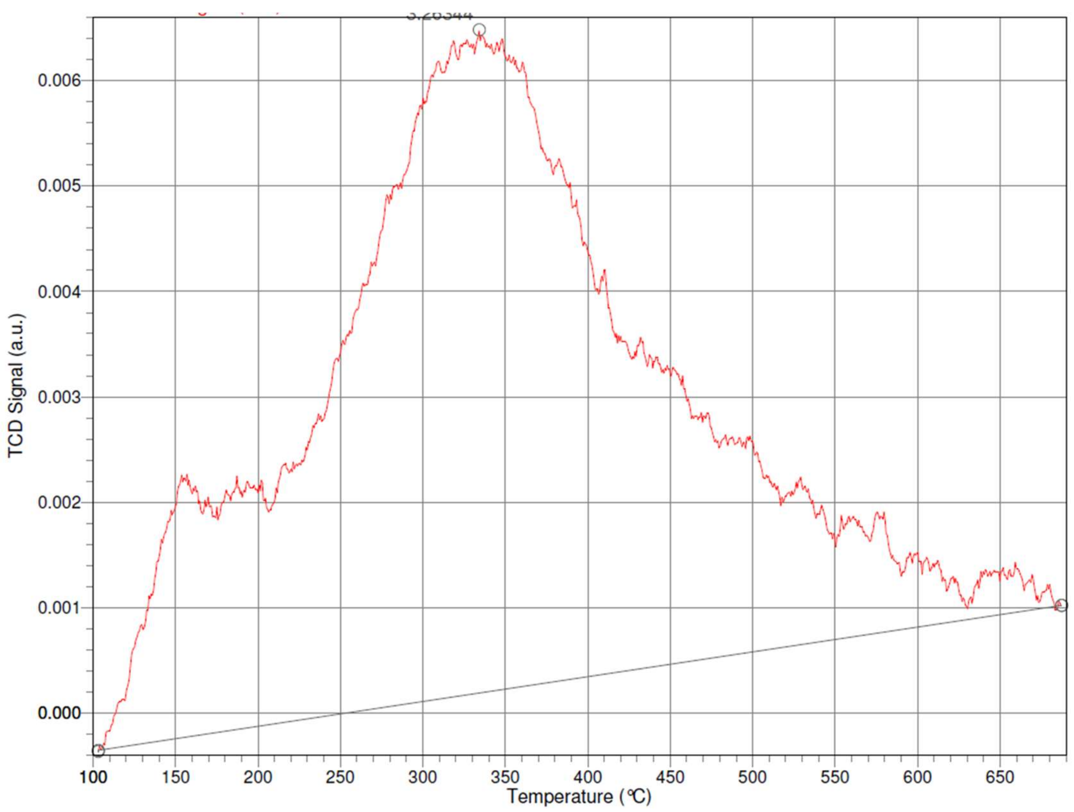

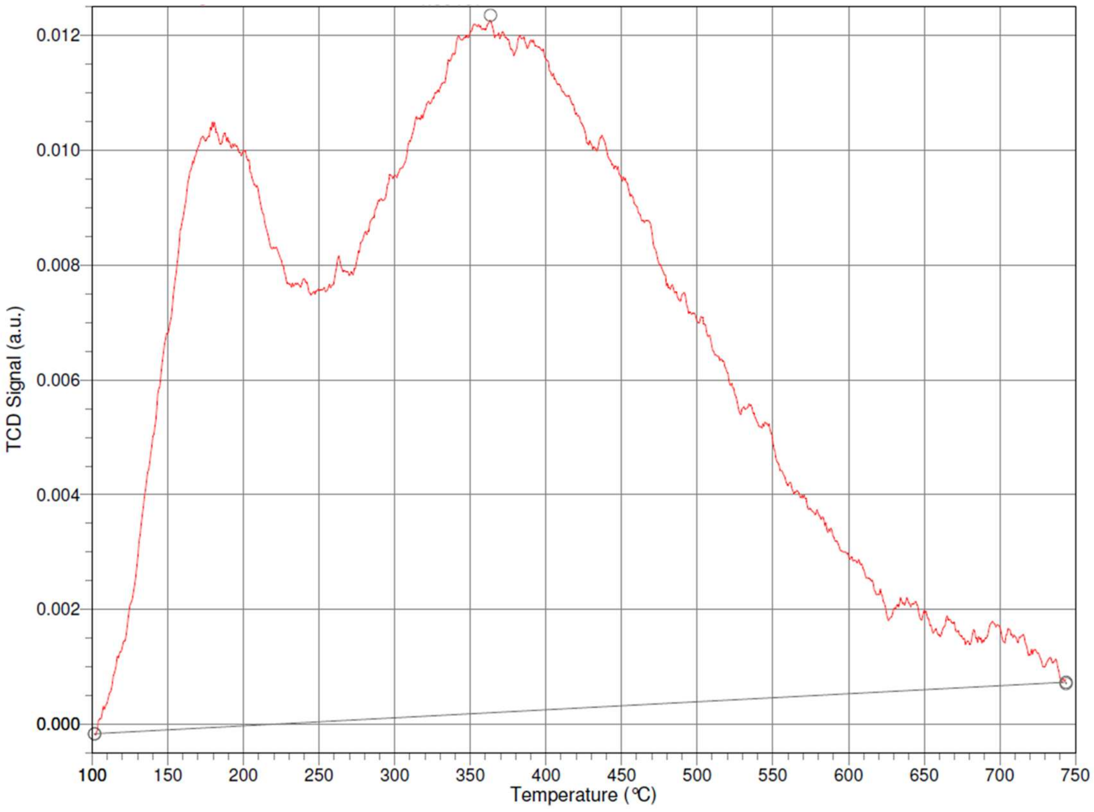

Figure S9. Temperature-programmed ammonia desorption (TPD) analysis of the H-Y zeolite (top, Si/Al = 15) and H-Beta zeolite (bottom, Si/Al = 12.5).

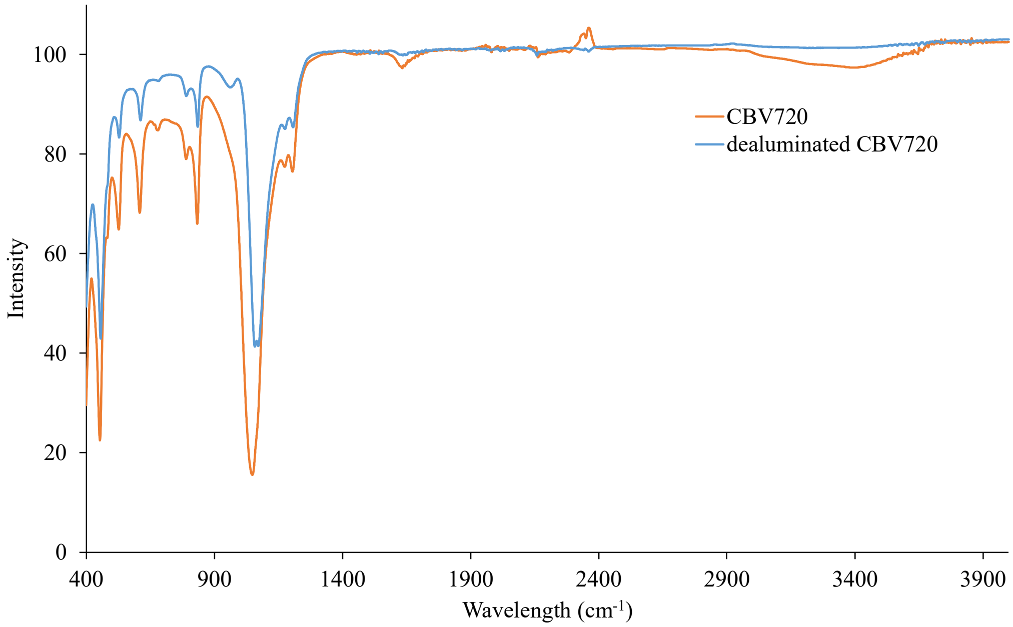

Figure S10. Fourier transform infrared spectroscopy (FT-IR) of the dealuminated H-Y zeolite compared to the starting H-USY zeolite (Si/Al = 15). Notice that the O-H band at 3000-3550 cm^{-1}, corresponding to the acid sites, decreases for the dealuminated H-Y zeolite. The zeolite sample name corresponds to the commercial notation.

Table S2. Physicochemical properties of the active acid zeolites employed in this work.

Solid Acid	Si/Al molar ratio	BET surface area ($m^2 \cdot g^{-1}$)[a]	Total pore volume ($cm^3 \cdot g^{-1}$)[a]	Particle or crystal size (µm)	Micropore volume ($cm^3 \cdot g^{-1}$)[a]	Mesopore volume ($cm^3 \cdot g^{-1}$)[a]
**H-Y (CBV720)**	15	723	0.44	0.70-1.00	0.32	0.13
**H-Y (CBV740)**	20	666	0.53	0.70-1.00	0.29	0.16
**H-Y (CBV760)**	28	753	0.52	0.70-1.00	0.33	0.14
**H-Y (CBV780)**	40	756	0.55	0.70-1.00	0.32	0.16
**H-Y (dealuminated)**	N.D.	667	0.51	0.70-1.00	0.24	0.20
**H-Beta**	12.5	566	0.89	0.6	0.19	0.30
**H-Mordenite**	7.6	416	0.35	-	0.17	-

[a] Brunauer, Emmett and Teller (BET) surface area and total pore volume values were determined by nitrogen adsorption-desorption experiments. N.D.: Non-determined. The zeolite sample names correspond to the commercial notation.

Table S3. Acidic properties of the zeolites employed in this work, determined from the corresponding FT-IR spectra after pyridine desorption.

		Brønsted acidity (µmol pyridine · g^{-1} catalyst)[a]				Lewis acidity (µmol pyridine · g^{-1} catalyst)[a]			
**Solid**	Si/Al	150 ºC	250 ºC	350 ºC	400 ºC	150 ºC	250 ºC	350 ºC	400 ºC
**H-Y (CBV-720)**	15	289.56	327.66	172.72	154.1	58.71	80.94	58.14	16.67
**H-Y (CBV-740)**	20	130.81	59.69	26.67	-	25.08	26.22	24.51	-
**H-Y (CBV-760)**	28	185.42	172.72	87.63	-	45.6	41.61	19.38	-
**H-Y (CBV-780)**	40	100.33	82.55	59.69	-	16.53	15.39	11.97	-
**H-Y (deAl)**	<1	41.61	8.55	8.55	-	-	-	-	-
**H-Beta**	12.5	374.65	278.13	154.94	-	393.3	372.21	324.90	-

[a] Measured by Fourier-transformed infrared spectroscopy (FT-IR) with adsorption and desorption of pyridine at different temperatures. Values of (µmol pyridine · g^{-1} of catalyst) were calculated using the corresponding extinction molar coefficients by Emeis.[S1] The zeolite sample names correspond to the commercial notation.

S2.6 ^{27}Al nuclear magnetic resonance (NMR).

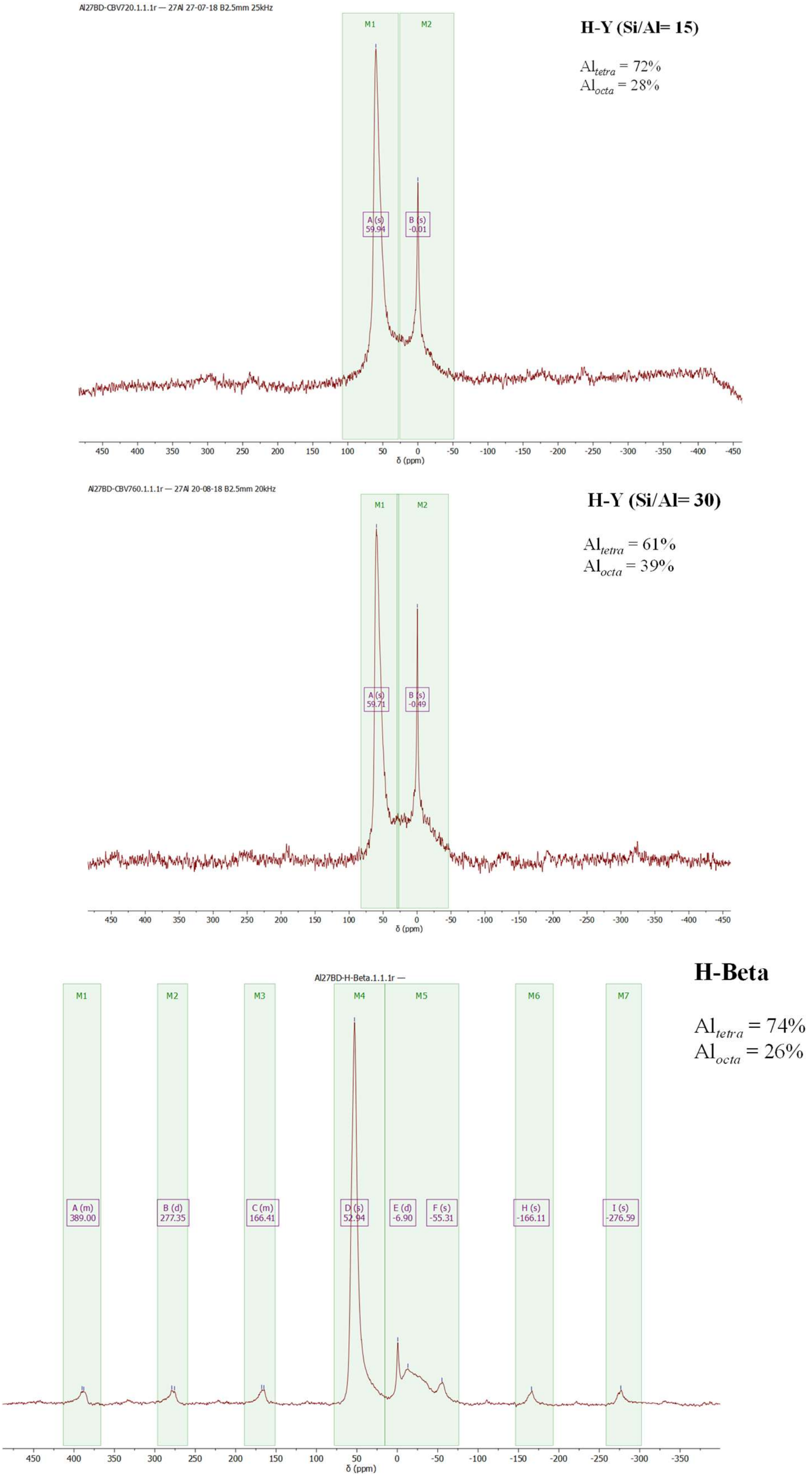

Figure S11. ^{27}Al solid-state nuclear magnetic resonance (ssNMR) of HY zeolite (top, Si/Al= 15; and middle, Si/Al= 30) and H-Beta zeolite (bottom, Si/Al= 12.5).

S3. Optimization of the intramolecular COM reaction with $FeCl_3$

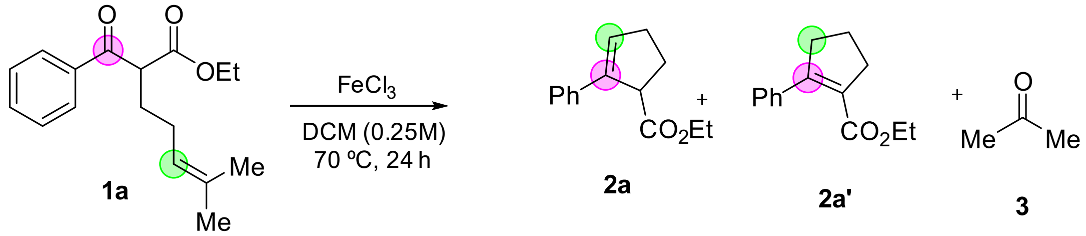

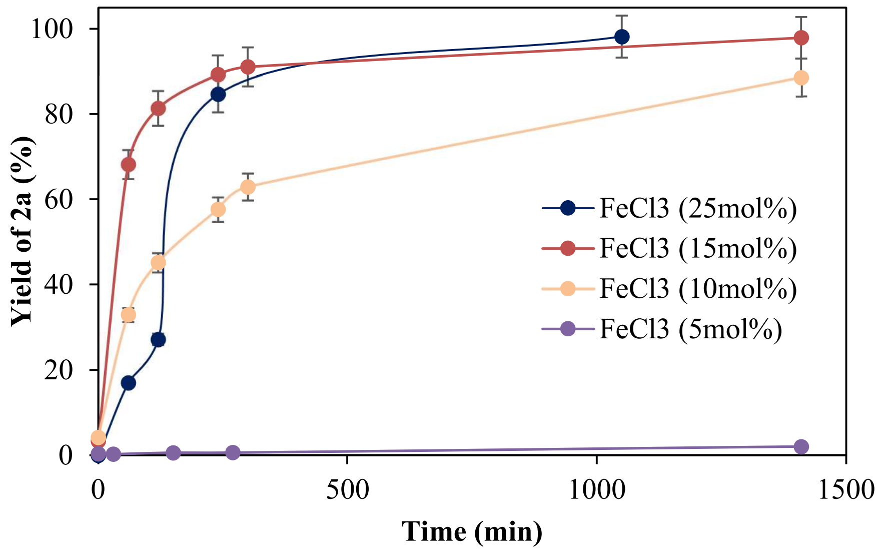

Figure S12. Kinetics for the optimization of the amount of $FeCl_3$ catalyst at 70 ºC. GC yields. Error bars account for a 5% uncertainty.

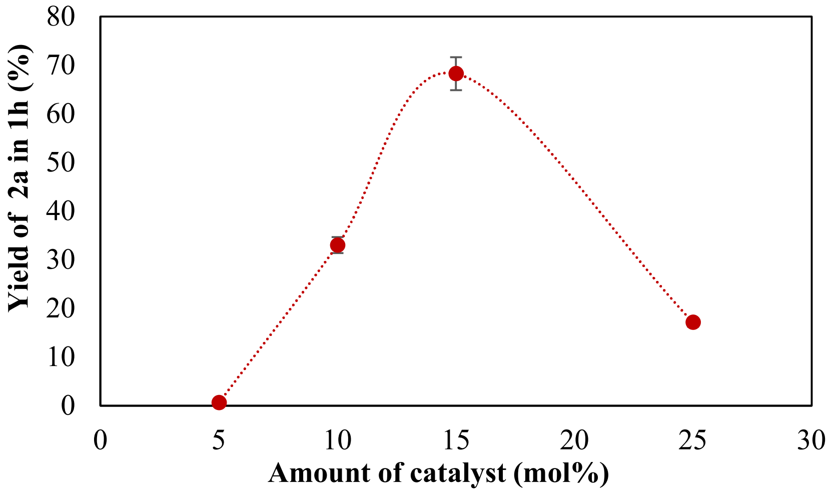

Figure S13. Yields obtained after 1 hour of reaction for each amount of $FeCl_3$ catalyst used. GC yields. Error bars account for a 5% uncertainty.

S4. Leaching and reusing of USY zeolites.

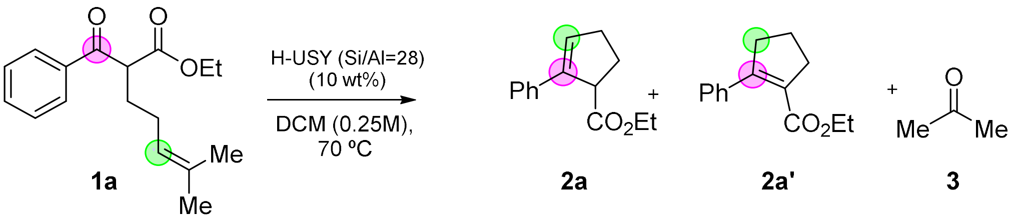

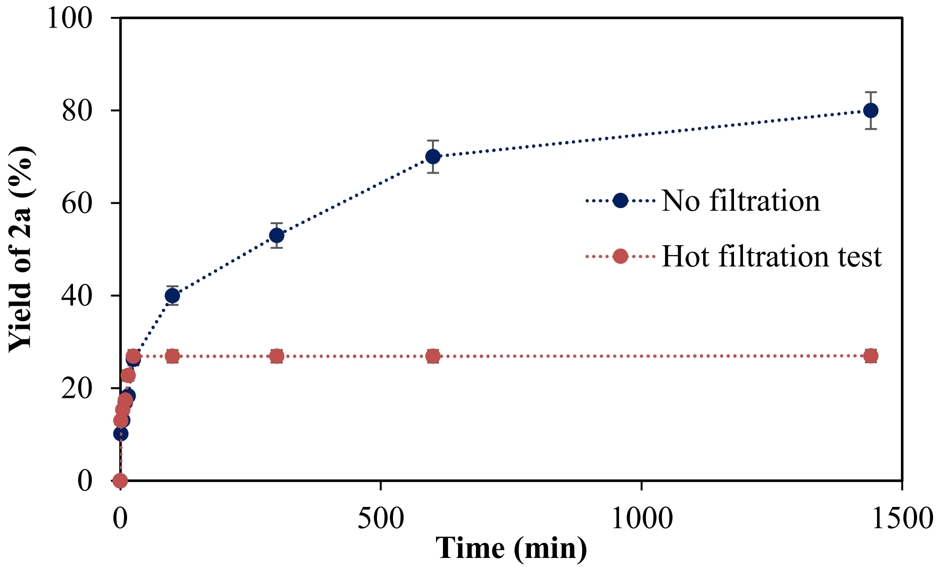

Figure S14. Hot filtration test for the carbonyl-olefin metathesis. GC yields. Error bars account for a 5% uncertainty.

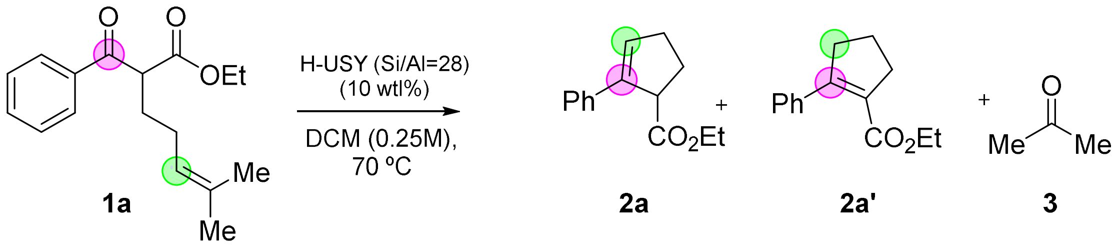

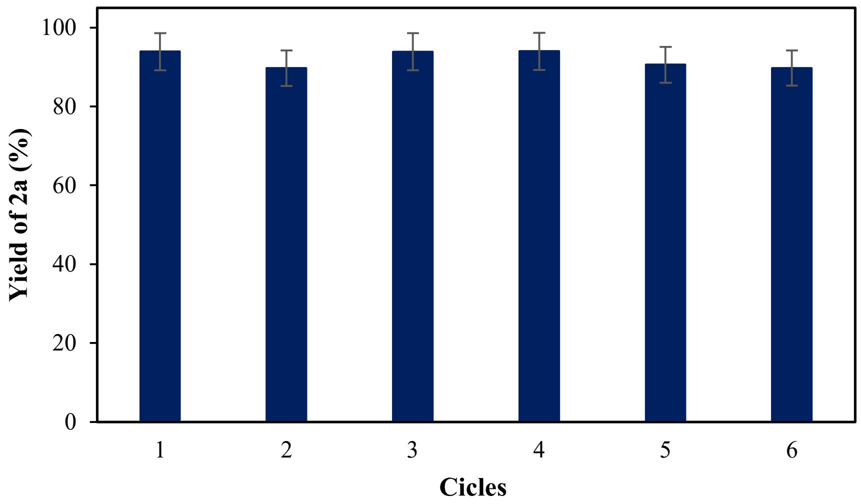

Figure S15. Reuses of the H-USY zeolite (Si/Al=28) under optimized reaction conditions for product **2a** (intramolecular carbonyl-olefin metathesis). GC yields. Error bars account for a 5% uncertainty.

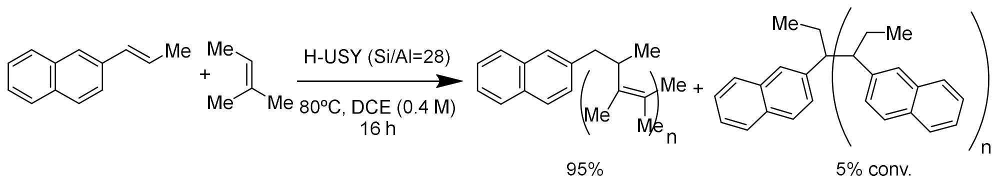

Figure S16. By-products observed for a typical intermolecular COM reaction.

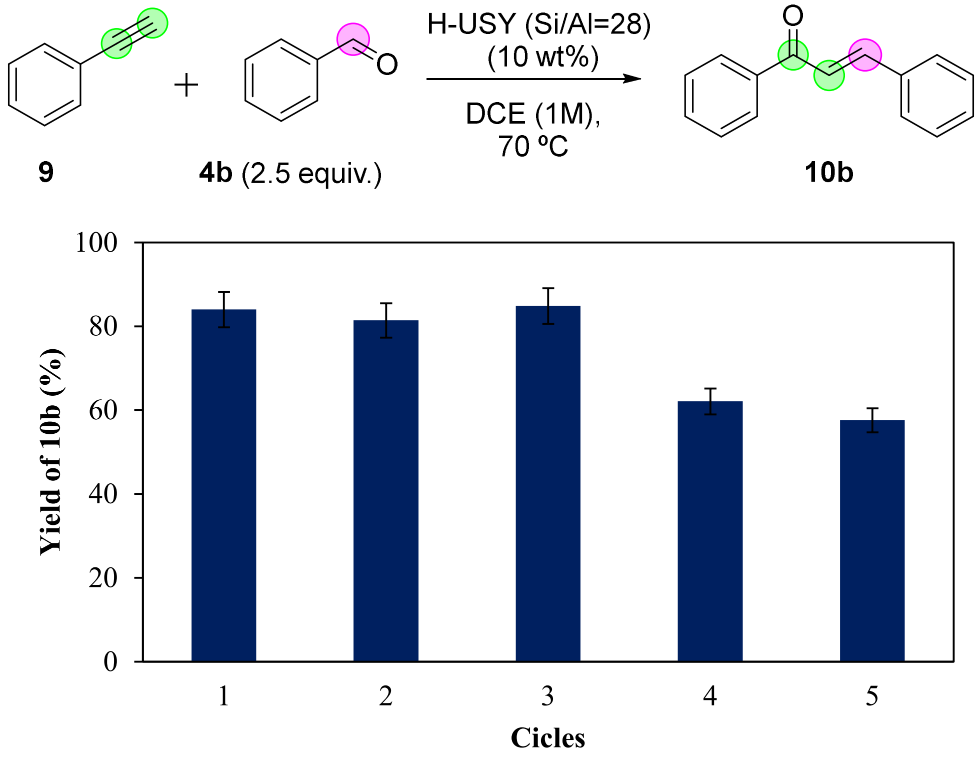

Figure S17. Reuse of H-USY zeolite (Si/Al=28) under the optimized reaction conditions for product **10b** (intermolecular carbonyl-alkyne metathesis). GC yields. Error bars account for a 5% uncertainty.

S5. Catalytic results with other solid acids.

S5.1. Catalyst screening and optimization.

Table S4. Results obtained for the intramolecular carbonyl-olefin metathesis using different sulfonic resins as catalysts. GC yields.

Entry	Catalyst	Amount of catalyst (mol%)	Conversion 24 h (%)
**1**	Nafion SAC-13 (wet)	5	1.1
**2**	Amberlyst A-15 (wet)	25	4.8
**3**	Amberlyst A-15 (dry)	50	15.5
**4**	Amberlyst A-16 (wet)	25	6.1
**5**	Amberlyst A-16 (dry)	50	8.6 (40.6 in 93 h)
**6**	Amberlyst BD20 (wet)	25	0.0

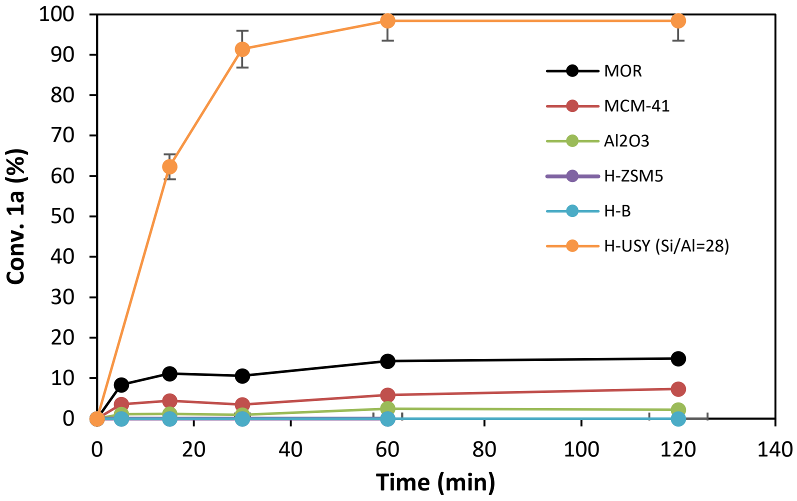

Figure S18. Kinetics for different aluminosilicates, as well as Al_2O_3. GC yields. Error bars account for a 5% uncertainty.

Table S5. Initial rates compared to the total amount of Brønsted acid sites obtained by (pyridine tritration) for each zeolite. GC results.

Zeolite	v_0 (% conv./min)	Funcionality (mmol H^+/g)
**H-USY (Si/Al=15)**	6.4759	0.274
**H-USY (Si/Al=20)**	0.4558	0.131
**H-USY (Si/Al=28)**	3.046	0.087
**H-USY (Si/Al=40)**	0.2332	0.087

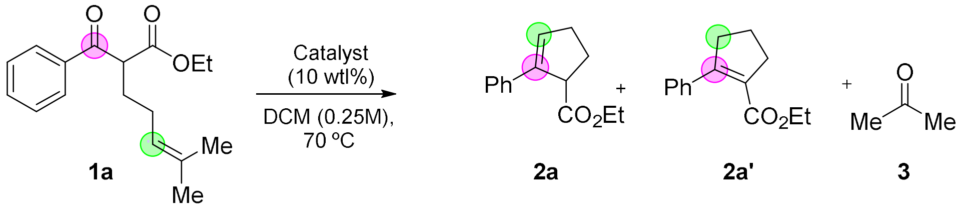

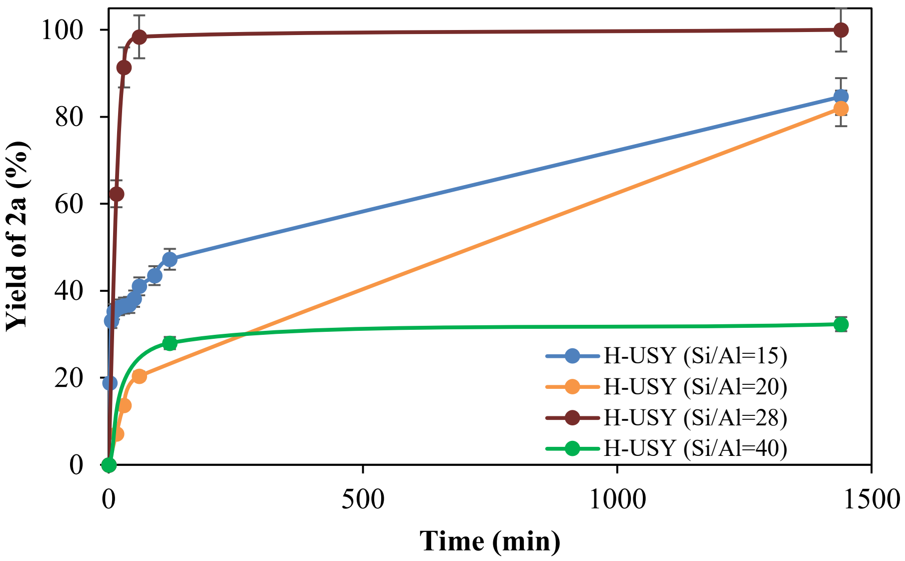

Figure S19. Kinetics for H-USY zeolite catalysts with different Si/Al ratio. GC yields. Error bars account for a 5% uncertainty.

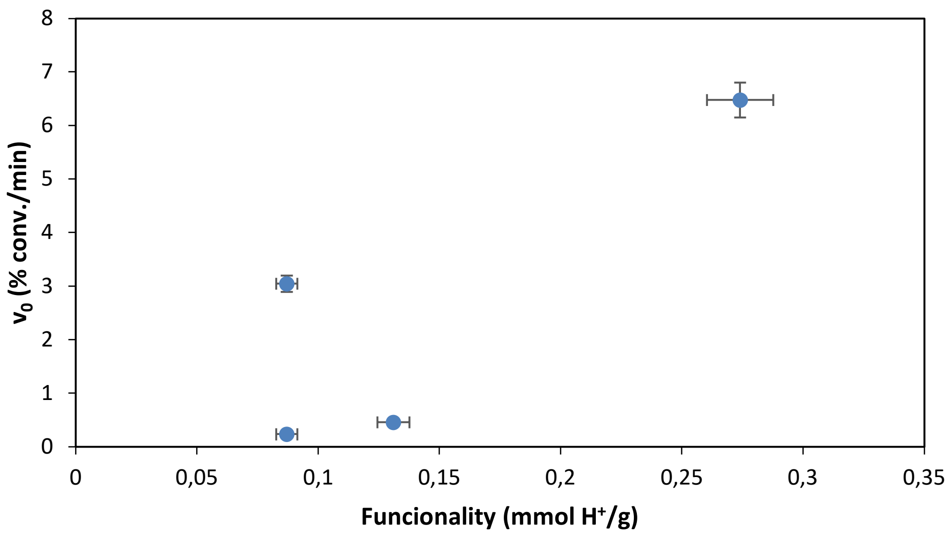

Figure S20. Lack of any clear correlation between the initial rate and the total amount of Brønsted acid sites (obtained by pyridine titration) of the zeolite catalyst. GC yields. Error bars account for a 5% uncertainty.

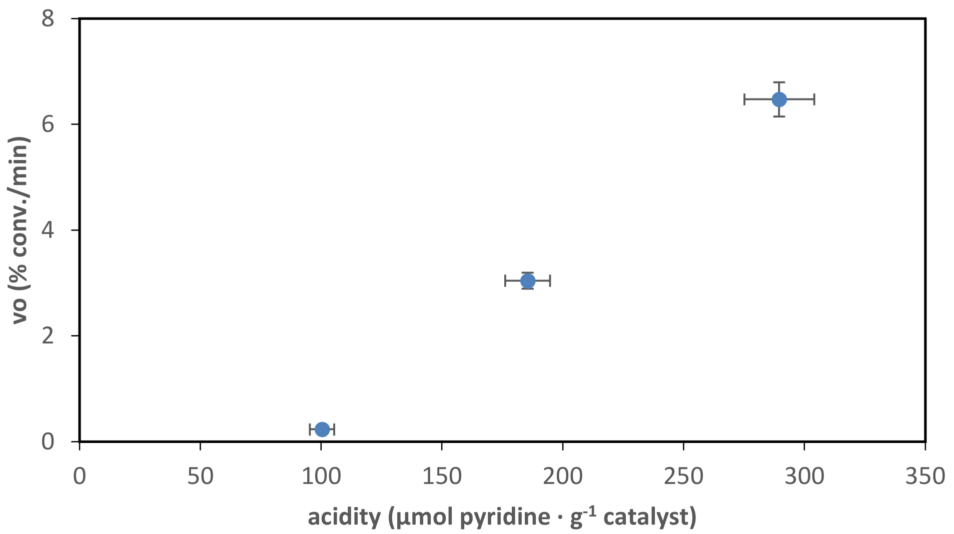

Figure S21. Correlation between the initial rate and the total amount of Brønsted acid sites after pyridine titration and desorption at 150 ºC over the zeolite catalyst. GC yields. Error bars account for a 5% uncertainty.

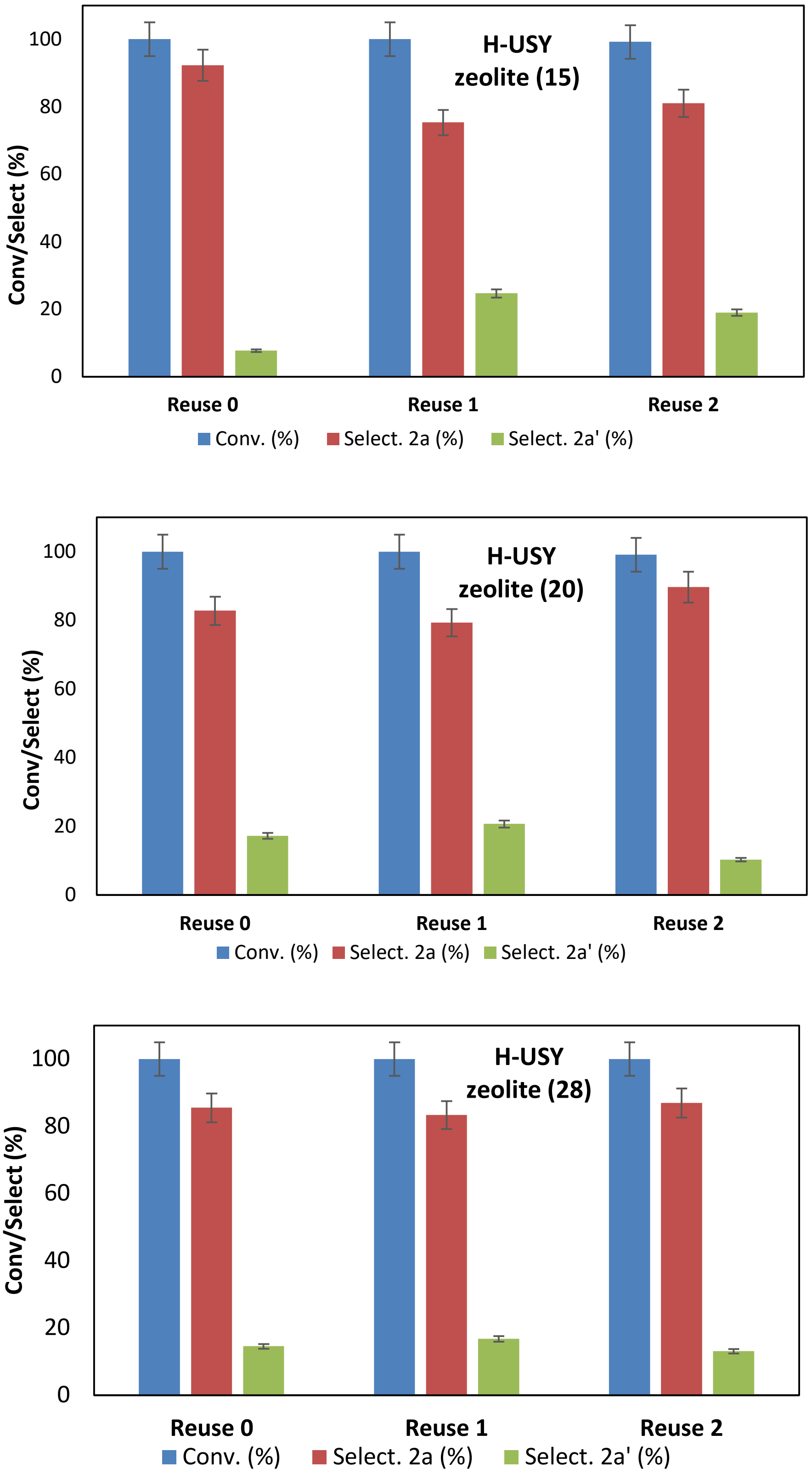

Figure S22. Comparison of three uses for different H-USY zeolites. GC yields. Error bars account for a 5% uncertainty.

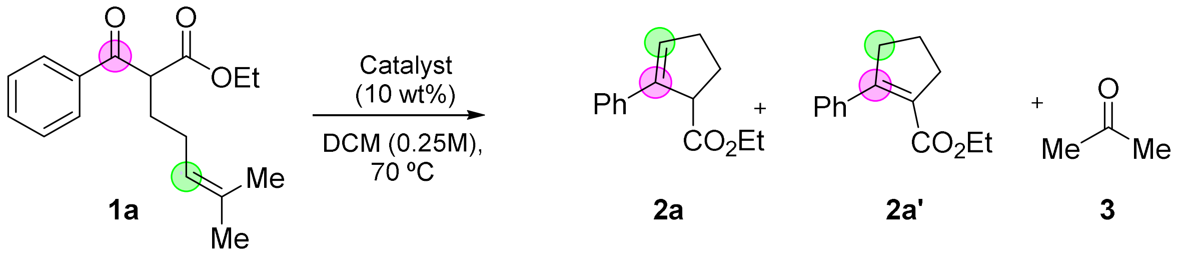

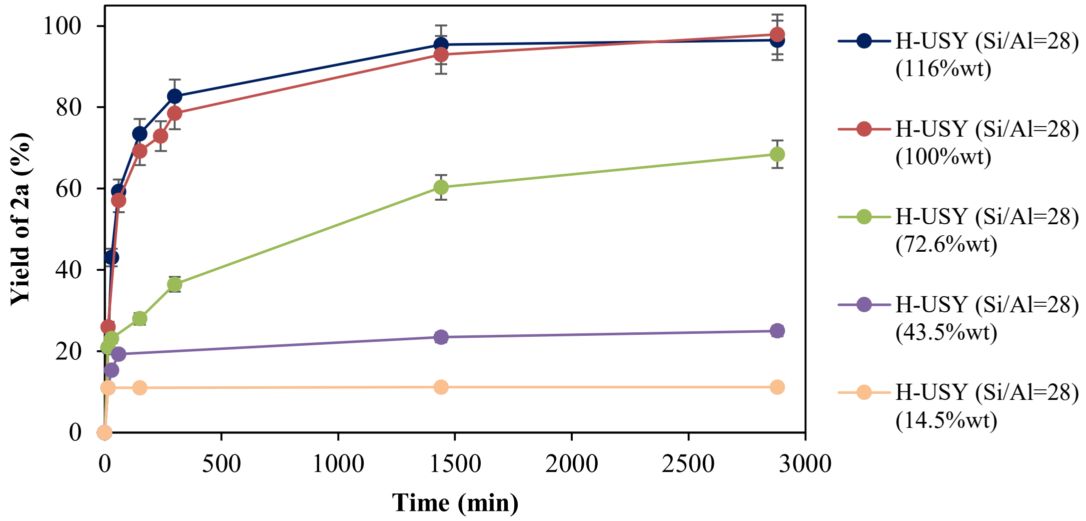

Figure S23. Optimization of the amount of catalyst (indicated respect to the reactant **1a**, ten times less respect to the reaction mixture) needed to carry out the carbonyl-olefin metathesis. GC yields. Error bars account for a 5% uncertainty.

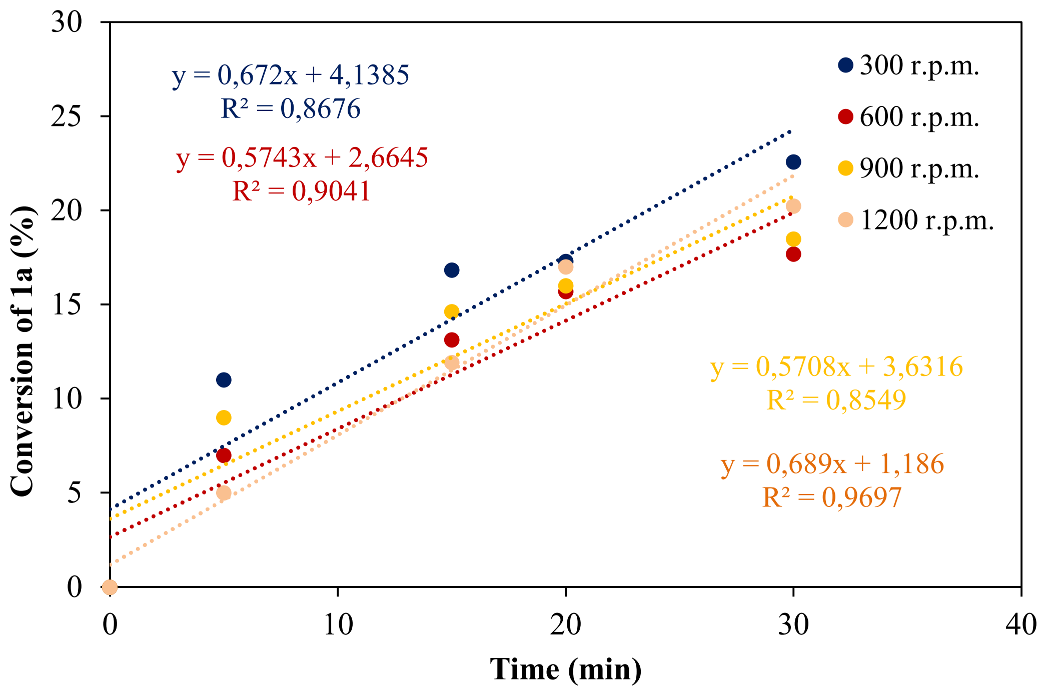

Figure S24. Kinetic plots for the intermolecular carbonyl-olefin metathesis reaction at different stirring velocities.

S5.2. Appearance of by-product 2a´.

Table S6. Results obtained for intramolecular COM reaction of **1a** using different homogeneous catalysts.[S2,S3]

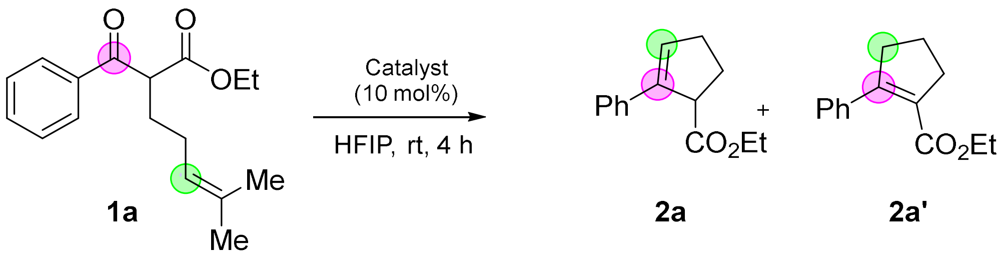

Entry	Catalyst	Yield of 2a	Yield of 2a' (%)	Total yield (%)
**1**	pTSA	80	7	87
**2**	Triflic	66	5	71
**3**	2,4-Dinitrobenzenesulfonic	75	4	79
**4**	2-Nitrobenzenesulfonic	89	4	93
**5**	4-Nitrobenzenesulfonic	85	5	90
**6**	4-(CF_3)benzenesulfonic	82	6	88
**7**	4-Acetylbenzenesulfonic	82	7	89
**8**	Benzenesulfonic	74	6	80
**9**	4-Dodecylbenzenesulfonic	76	6	82
**10**	4-Hydroxybenzenesulfonic	75	7	82
**11**	10-Camphorsulfonic	80	5	85
**12**	Methylsulfonic	78	10	88
**13**[b]	$HNTf_2$	76	6	82

S6. Optimization of the intermolecular COM reaction.

S6.1. Catalyst screening for the intermolecular carbonyl-olefin metathesis.

Table S7. Catalyst screening for the intermolecular carbonyl-olefin metathesis. GC yields.

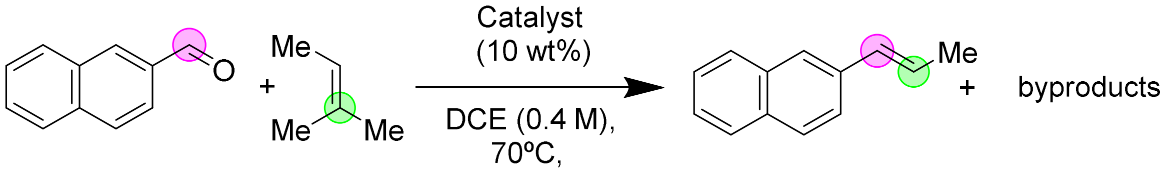

4a (0.2 mmol) **5a** (2 equiv.) **6aa**

Entry	Catalyst	Conv. (%)	Select. Prod. (%)	Select. byprod. (%)
**1**	H-USY zeolite (Si/Al 15) (dry)	82	42	58
**2**	H-USY zeolite (Si/Al 28) (dry)	93	41	59
**3**	H-USY zeolite (Si/Al 40) (wet)	90	44	56
**4**	Amberlyst A-15	62	24	76
**5**	Amberlyst A-16	41	29	70
**6**	Amberlyst BD20	13	17	83
**7**	Nafion™	42	35	65

S6.2. Solvent screening.

Table S8. Solvent screening for the intermolecular carbonyl-olefin metathesis. GC yields.

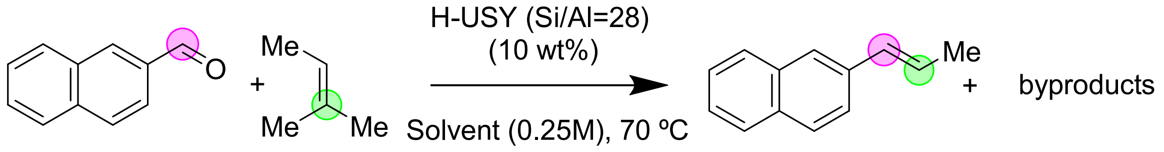

4a (0.2 mmol) **5a** (2 equiv.) **6aa**

Entry	Solvent	Equiv. aldehyde	Equiv. alkene	Conv. (%)	Select. (%)	Select. Subprod. (%)
**1**	**DCM**	**1**	**5**	**98.3**	**42.6**	**57.4**
**2**	**DCM**	**1**	**2**	**71.0**	**69.1**	**30.9**
**3**	Dioxane	1	5	97.5	37.8	62.2
**4**	Dioxane	1	2	40.3	43.2	56.8
**5**	Chloroform	1	5	92.0	48.7	51.3
**6**	Chloroform	1	2	63.9	42.4	57.6
**7**	ACN	1	5	4.3	99.9	0.0
**8**	DCE	1	2	85.6	48.6	51.4
**9**	Hexane	1	2	63.1	56.5	43.5
**10**	THF	1	2	2.6	99.9	0.0
**11**	DMF	1	2	0.5	-	-
**12**	**DMSO**	**1**	**5**	**93.4**	**44.1**	**55.9**
**13**	DMSO	1	2	0.0	-	-

S6.3. Solvent amounts screening.

Table S9. Optimization of the amount of solvent required for the intermolecular carbonyl-olefin metathesis reaction. GC yields.

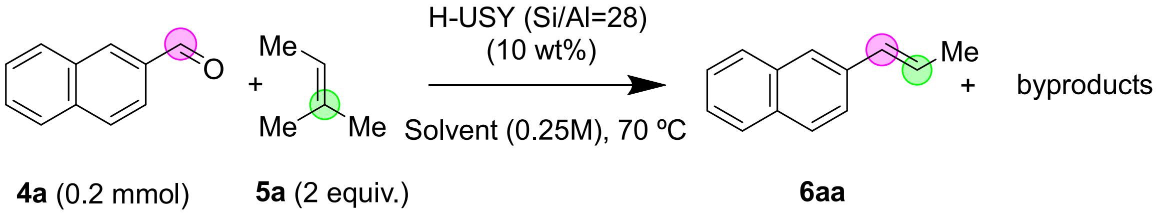

Entry	Conc. [M]	Conv. (%)	Select. (%)	Select. Subprod. (%)
**1**	0.4	71.0	69.1	30.9
**2**	1.0	85.8	54.4	45.7
**3**	Solvent free	78.9	69.2	30.8
**4**[a]	Solvent free	0.0	-	-

S6.4. Zeolite amount.

Table S10. Optimization of the amount of zeolite required for the intermolecular carbonyl-olefin metathesis reaction. GC yields.

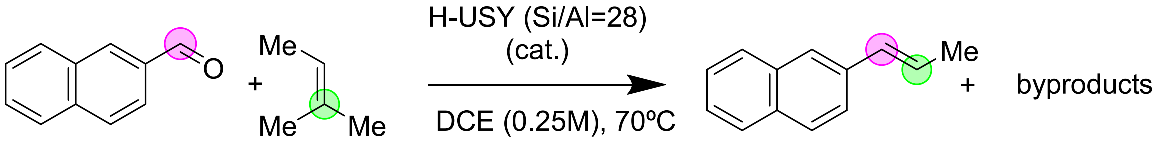

4a (0.2 mmol) **5a** (2 equiv.) **6aa**

**Entry**	**H-USY (Si/Al = 28) (mg)**	**Equiv. alkene**	**Conv. (%)**	**Select (%)**	**Select. Subprod. (%)**
**1**	50	2	71.0	69.1	30.9
**2**	20	2	64.6	49.7	50.3
**3**	10	2	44.2	45.2	54.8
**4**	5	2	28.7	47.9	52.0
**5**	0	2	0.0	-	-

S7. Optimization of the intermolecular CAM reaction.

Table S11. Optimization of the solvent needed to perform the intermolecular carbonyl-alkyne metathesis. GC yields.

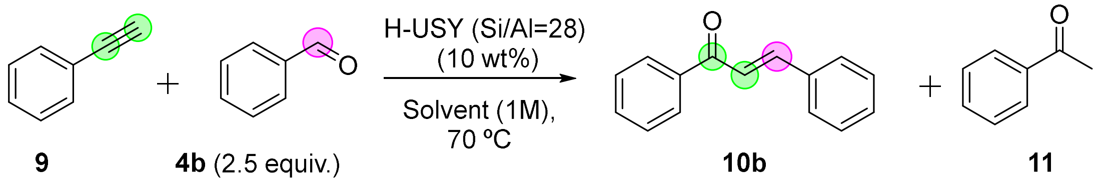

Entry	Solvent	Conc. [M]	Yield 10%	Yield 11 (%)
**1**	MeOH	1.00	59.3	29.4
**2**	MeOH	0.50	40.1	39.2
**3**	MeOH	0.25	21.5	42.7
**4**	-	-	68.4	28.4
**5**	DCM	1.00	61.4	37.3
**6**	DCM	0.50	57.8	40.5
**7**	DCM	0.25	51.4	45.3

Table S12. Optimization of the equivalents needed to perform the intermolecular carbonyl-alkyne metathesis. GC yields.

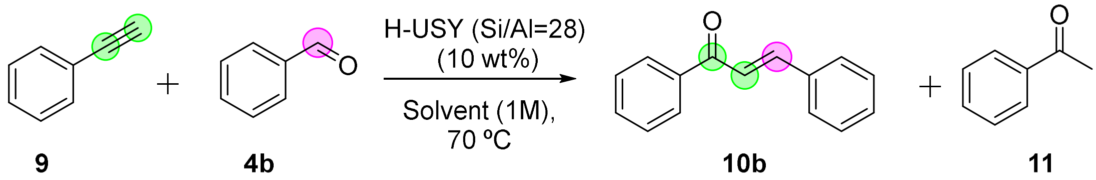

Entry	Aldehyde 4 (mmol)	Alkyne 9 (mmol)	Solvent	Yield 10 (%)	Yield 11 (%)
1	0.250	0.625	MeOH (1M)	82.4	-
**2**	0.250	0.625	-	40.0	-
**3**	0.625	0.250	MeOH (1M)	59.3	29.4
**4**	0.250	0.250	MeOH (1M)	31.2	64.6
**5**	0.250	0.375	-	81.3	-

Table S13. Optimization of the amount of catalyst needed to perform the intermolecular carbonyl-alkyne metathesis. GC yields.

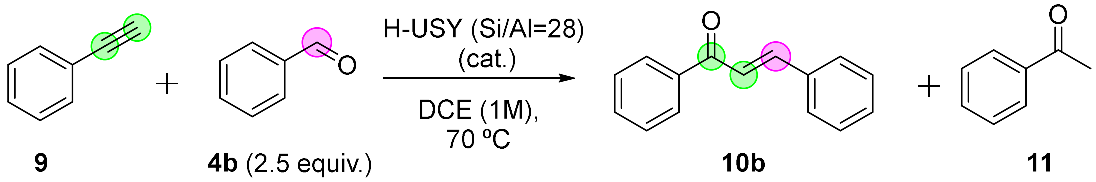

Entry	Catalyst (mg)	Yield 10 (%)	Yield 11 (%)
**1**	30	82.4	**-**
**2**	20	77.2	-
**3**	10	68.4	-

S8. Comparison of the results with reported reactions.

Table S14. Comparison of the results obtained with reported procedures using homogeneous catalysts.

Entry	Product	Yield obtained	Previous Reaction Conditions	Reference
**1**	**2a**	99%	$FeCl_3$ (5 mol%), DCE 0.01M, rT	99%[S4]
**2**	**2b**	82%	$FeCl_3$ (5 mol%), DCE 0.01M, rT	72%[S4]
**3**	**8**	74%	$AgSbF_6$ (10 mol%) DCE, 80 ºC	93%[S5]
			$BF_3(OEt_2)$ (20 mol%) DCE, 50 ºC	66%[S5]
			HBF_4 (20 mol%) DCE, 50 ºC	61%[S5]
**4**	**6aa**	50%	$TrBF_4$ (20 mol%), DCM (0.3 M), rT	60%[S6]
**5**	**6ba**	25%	$TrBF_4$ (20 mol%), DCM (0.3 M), rT	73%[S6]
**6**	**6fa**	42%	$TrBF_4$ (20 mol%), DCM (0.3 M), rT	54%[S6]

S9. Nature of the catalytic sites.

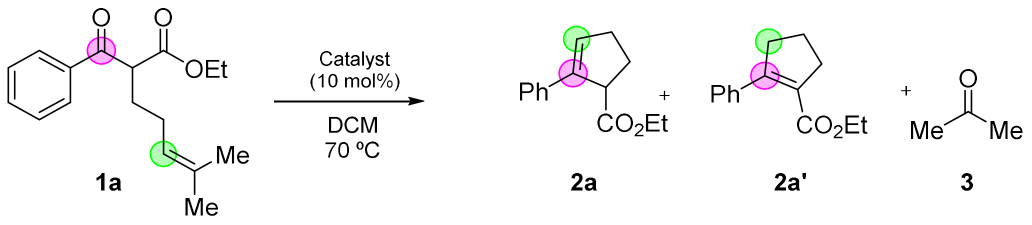

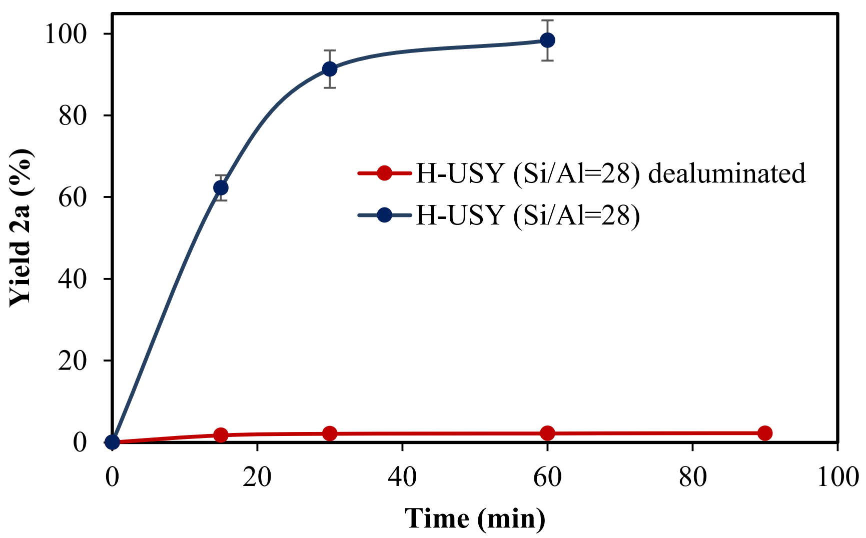

Figure S25. Kinetics for H-USY zeolite used and dealuminated. Error bars account for a 5% uncertainty.

Table S15. Inductively coupled plasma-atomic emission spectroscopy (ICP-AES) results for the H-Y zeolite (Si/Al= 15). The zeolite sample name corresponds to the commercial notation.

CBV720 H-Y sample	Concentration average (ppm)
**Pt**	10.44
**Cr**	1.19
**Ni**	3.17
**Co**	<0.01
**Ru**	<0.01
**Au**	9.47
**Mn**	0.64
**Fe**	146.25
**Cu**	11.65
**Ag**	0.95
**Pd**	<0.01
**Rh**	<0.01
**Al**	30874.97

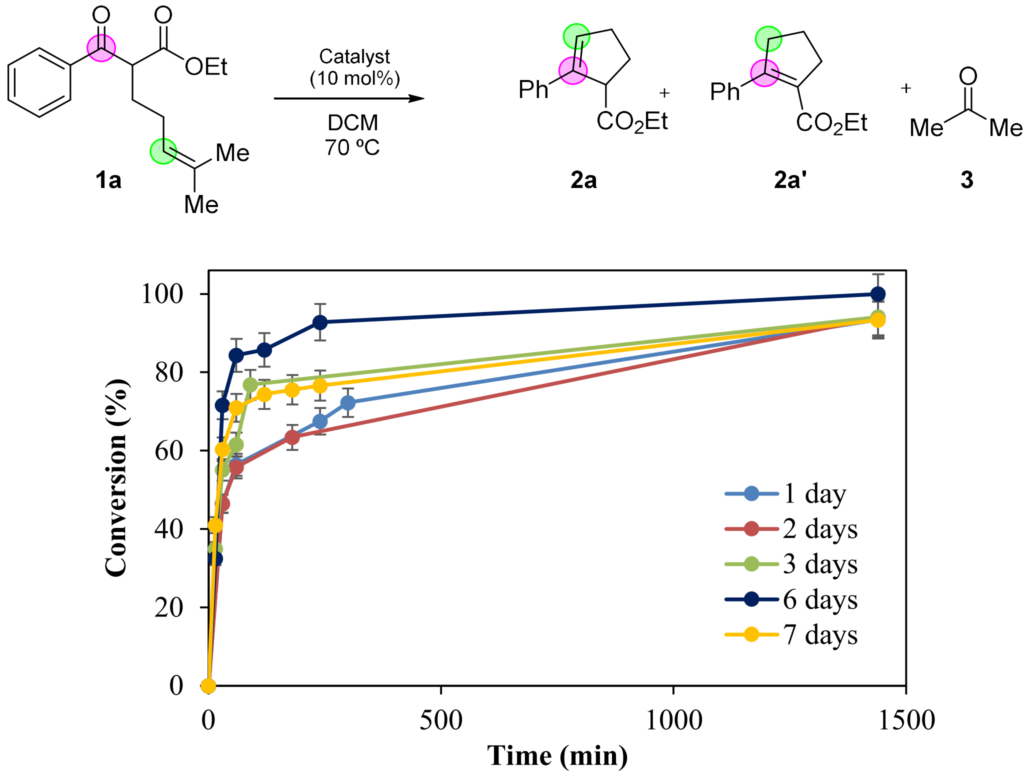

Figure S26. Kinetic experiment to observe the different activity when zeolite is open to ambient conditions. Error bars account for a 5% uncertainty.

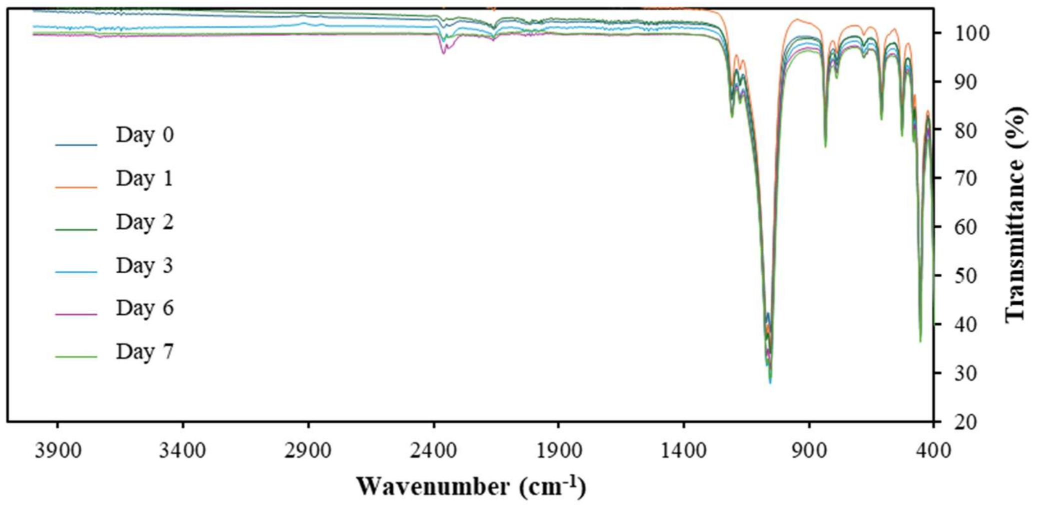

Figure S27. FT-IR spectroscopy of the H-USY zeolite during a week.

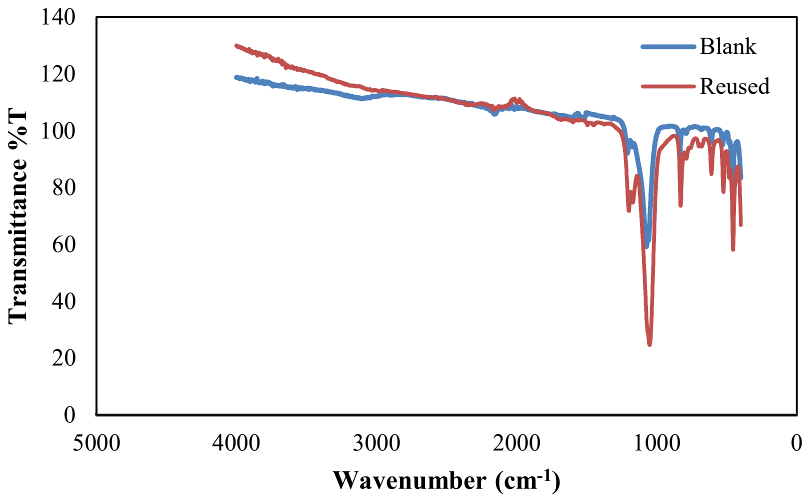

Figure S28. FT-IR spectroscopy of the H-USY zeolite before and after being used for performing carbonyl-alkyne metathesis reaction.

Table S16. Results obtained for the carbonyl-alkyne metathesis reaction using different conditions.

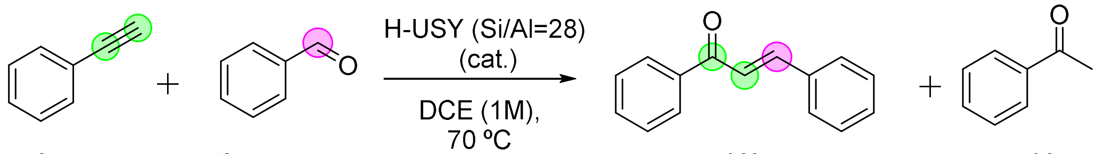

9 **4b** (2.5 equiv.) **10b** **11**

Entry	Conditions	Yield 10b (%)	Yield 11 (%)
**1**	N_2 atmosphere	48.8	18.1
**2**	Molecular Sieves	19.2	25.5
**3**	Benzaldehyde water absent	59.4	23.9

S10 Reaction Mechanism.

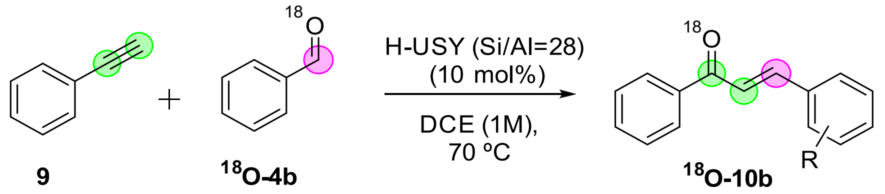

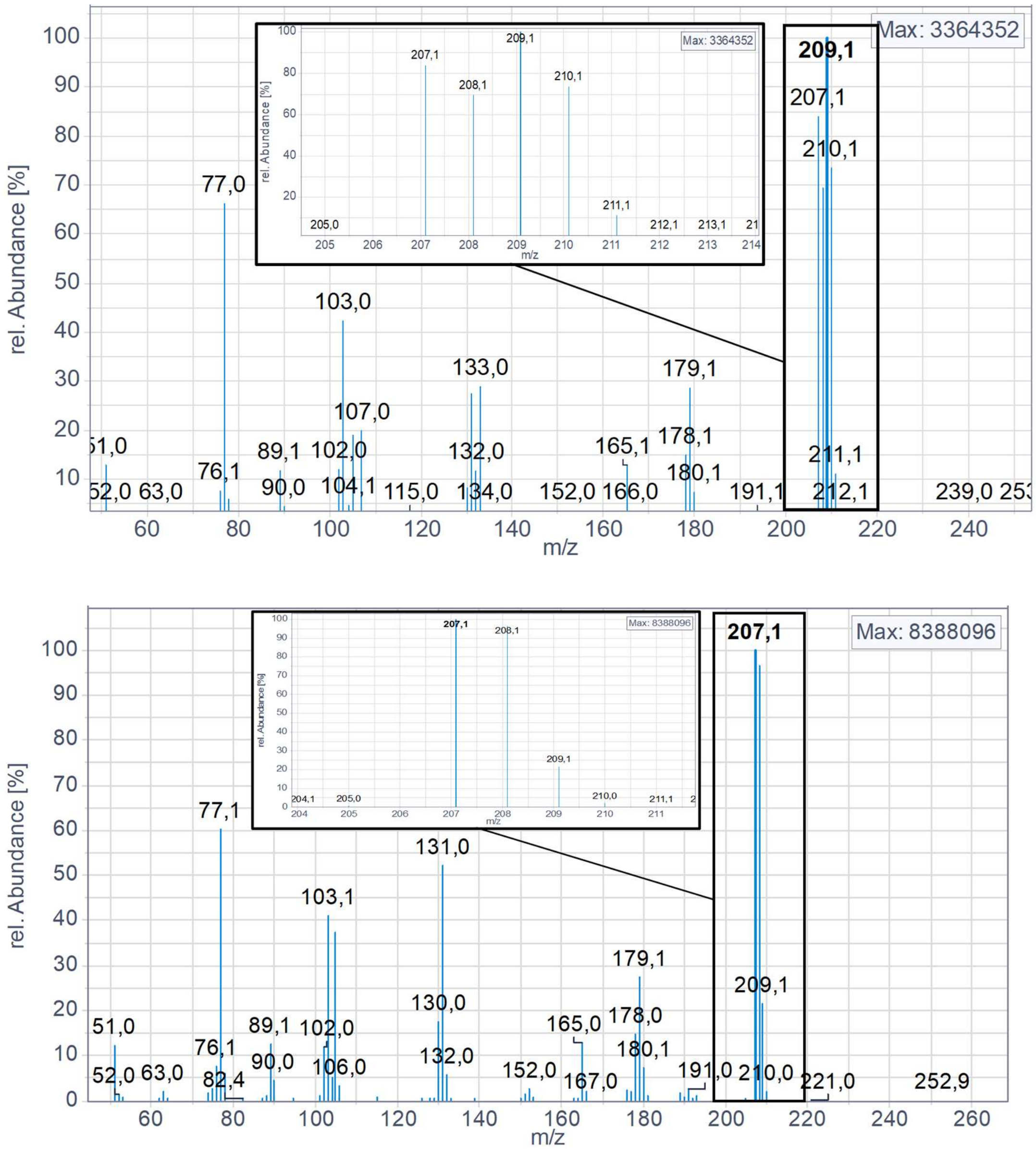

Figure S29. GC-MS of the corresponding ^{18}O-chalcone product (**^{18}O-10b**, top) and GC-MS of the corresponding chalcone without ^{18}O (**10b**, bottom).

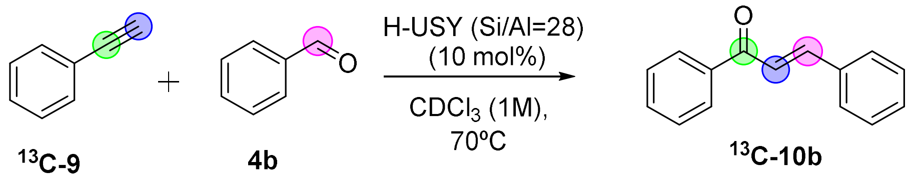

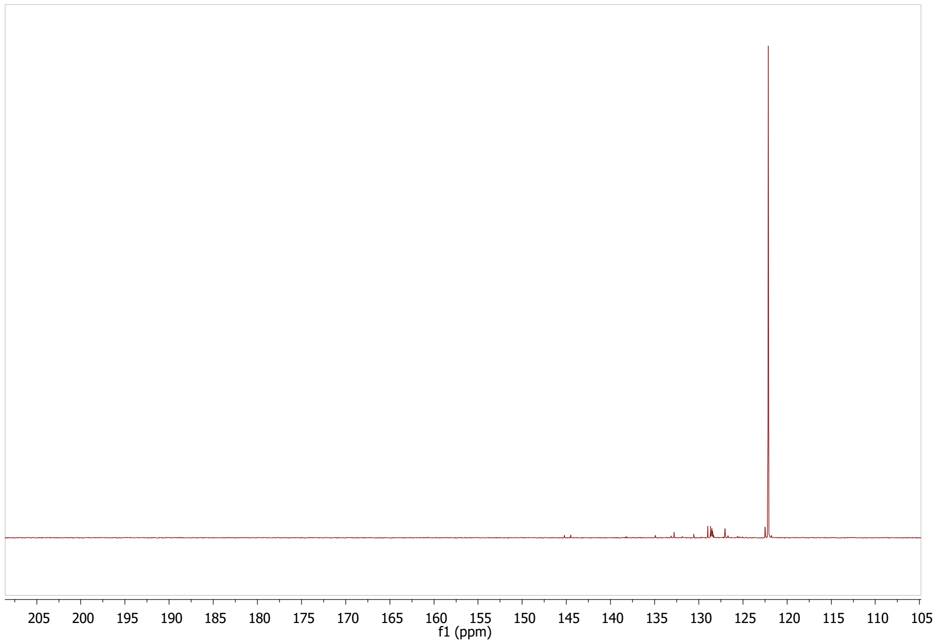

Figure S30. Diagnostic area of the ^{13}C NMR spectrum from the chalcone product **^{13}C-10b** (^{13}C marked in blue, 121 ppm).

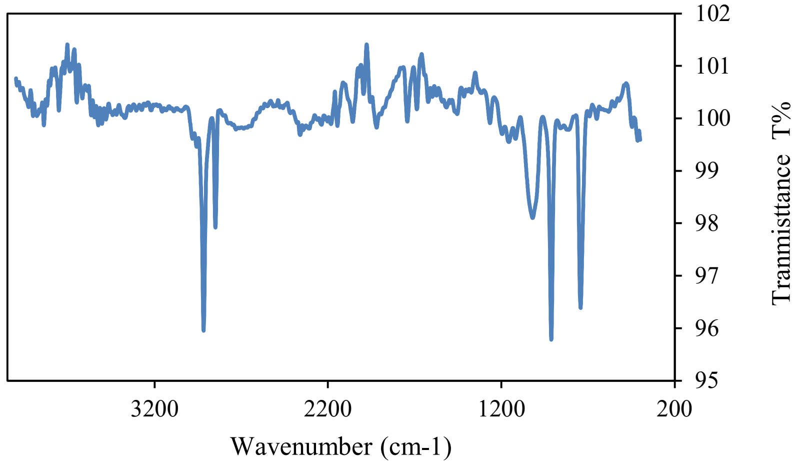

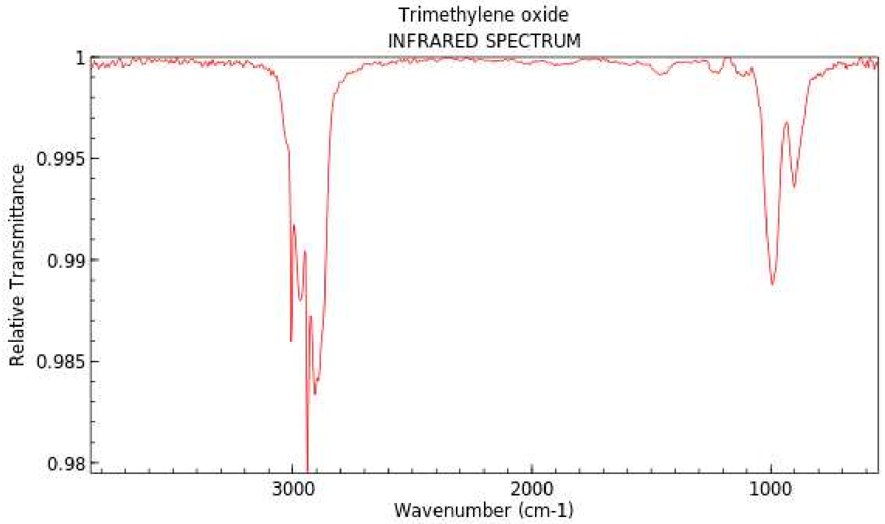

Figure S31. Comparison between the FT-IR spectrum obtained for the oxetane **Int** (top) and the canonical trimethylene oxide oxetane (bottom).

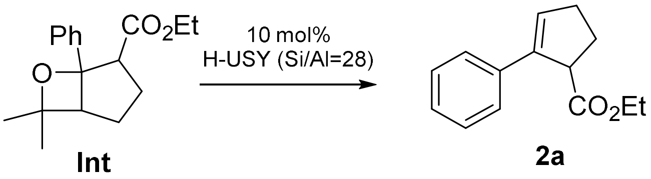

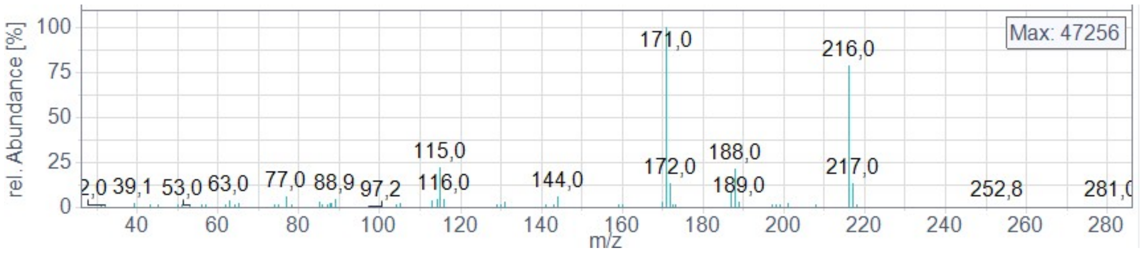

Figure S32. GC-MS spectra of **Int-1** (top) and the corresponding product **2a** (bottom) after making react the oxetane **Int-1** under the optimized reaction conditions.

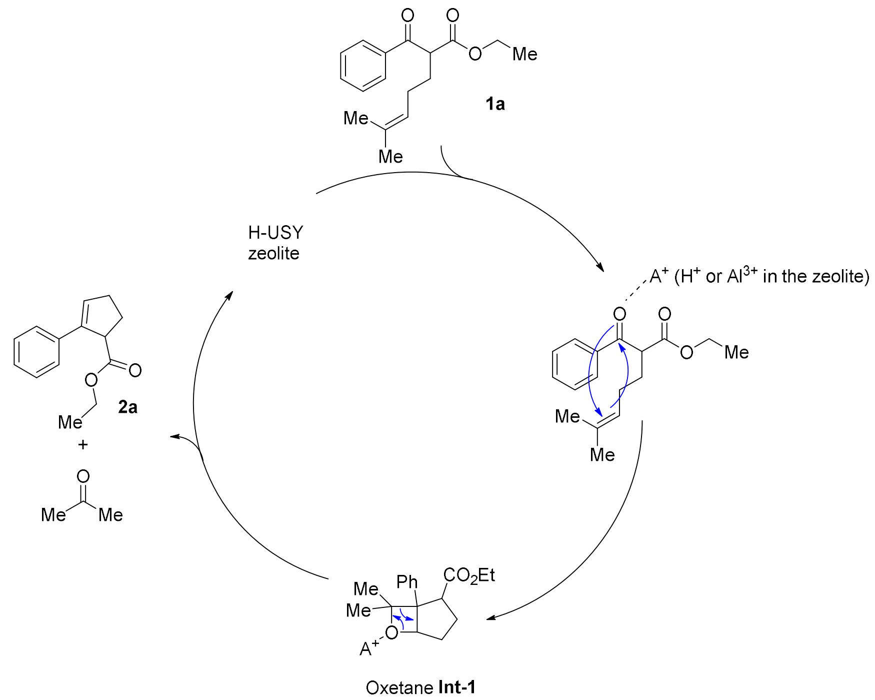

Figure S33. Proposed reaction mechanism for the zeolite-catalyzed COM reaction of **1a**.

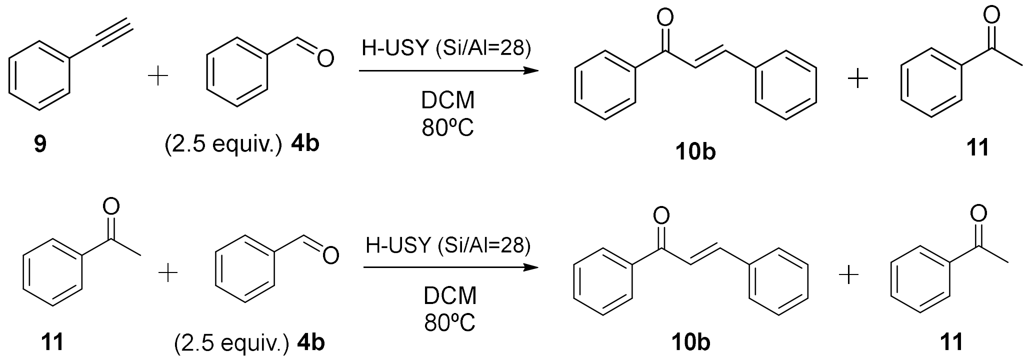

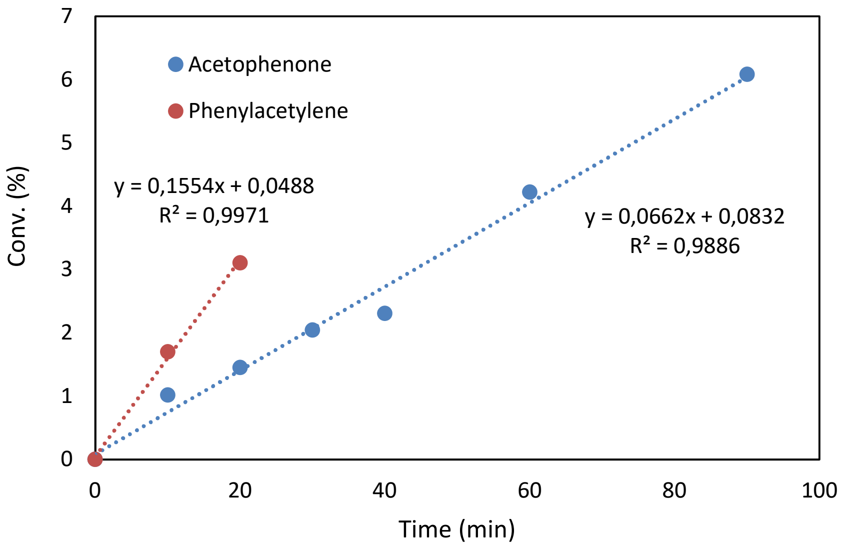

Figure S34. Comparative kinetic results for the CAM reaction of **9** and **4b** with the aldol condensation between acetophenone **11** and benzaldehyde **4b**, under identical reaction conditions.

Additional references.

(S1) Emeis, C. A. Determination of integrated molar extinction coefficients for infrared absorption bands of pyridine adsorbed on solid acid catalysts. *J. Catal.* **1993**, 141, 347–354. https://doi.org/https://doi.org/10.1006/jcat.1993.1145;

(S2) To, T. A.; Pei, C.; Koenigs, R. M.; Nguyen, T. V. Hydrogen Bonding Networks Enable Brønsted Acid-Catalyzed Carbonyl-Olefin Metathesis. *Angew. Chem. Int. Ed.* **2022**, *61*, e202117366.

(S3) To, T. A.; Mai, B. K.; Nguyen, T. V. Toward Homogeneous Brønsted-Acid-Catalyzed Intramolecular Carbonyl–Olefin Metathesis Reactions. *Org. Lett.* **2022**, *24*, 7237–7241.

(S4) Ludwig, J. R.; Zimmerman, P. M.; Gianino, J. B.; Schindler, C. S. Iron(III)–catalysed carbonyl–olefin metathesis. *Nature* **2016**, *533*, 374–379.

(S5) Rhee, J. U.; Krische, M. J. *Org. Lett.* **2005**, *7*, 2493–2495.

(S6) Ludwig, J. R.; Schindler, C. S. *Synlett* **2017**, *28*, 1501–1509.
